# Development and Characterization of Thermoplastic Composites Based on Recycled HDPE from Railway Sleepers’ Fastening Bushes and Scraped Fractions from Carbon Fiber Waste Upcycling

**DOI:** 10.3390/polym18111309

**Published:** 2026-05-26

**Authors:** Roberto Petrucci, Marco Rallini, Maurizio Natali, Luigi Torre

**Affiliations:** Department of Civil and Environmental Engineering, University of Perugia, Strada di Pentima 4, 05100 Terni, Italy; marco.rallini@unipg.it (M.R.); maurizio.natali@unipg.it (M.N.); luigi.torre@unipg.it (L.T.)

**Keywords:** extrusion compounding, recycled high-density polyethylene, recycled short carbon fibers, environmental sustainability, mechanical properties, electric conductivity, scanning electron microscopy, life cycle analysis

## Abstract

The railway sector is crucial for transportation, but infrastructure maintenance generates significant waste and requires large amounts of materials, increasing environmental impact. Circular economy integration mitigates this impact through material recovery. This study focused on the recycling of bushes embedded in railways sleepers, currently disposed of in landfills, obtaining high-density polyethylene (HDPE). The developed scalable process converted contaminated bushes into pellets, whose environmental sustainability was assessed through life cycle analysis. Challenges of the recycled material, such as high viscosity and heterogeneity, were partially addressed with a slipping agent and a compatibilizer, increasing the material melt index from 0.71 to 1.62 g/10 min. Carbon fiber waste addition improved thermal stability, mechanical stiffness, and electrical conductivity. Compatibilized blends offered the best balance of mechanical properties but lower electrical conductivity. The Young modulus was increased from 1.20 GPa for the neat matrix to 4.40 GPa for the system containing 30% carbon fibers in weight, with no significant decreases in the yield stress, while showing the lowest electrical conductivity. To reduce environmental impact and produce a tougher material without compromising conductivity, the compatibilizer was replaced with HDPE from PET bottle caps, resulting in comparable mechanical properties and higher electrical conductivity but reduced fiber/matrix interface.

## 1. Introduction

Among the various forms of sustainable transportation, the railway sector plays a crucial role, and the integration of circular economy principles into railway infrastructure offers significant potential to enhance environmental performance [1]. In Italy, railway lines include millions of reinforced concrete sleepers (estimated between 55 and 70 million units), corresponding to approximately 420–500 tons per kilometer. These components often incorporate high-density polyethylene (HDPE) bushes embedded in the concrete, acting as damping elements within fastening systems.

Although sleepers are designed for a service life of 30–50 years, they are frequently replaced during maintenance operations—sometimes every five years—for safety reasons.

As a result, ballasted tracks and crossbeams represent critical end-of-life infrastructure components whose management requires improved circular strategies. While steel elements in rails and fasteners are fully recyclable, the recycling of concrete and polymeric fractions remains less developed due to economic and regulatory constraints. Consequently, large quantities of inert materials and plastic components are currently landfilled, despite their high performance.

Previous studies have demonstrated that materials recovered from decommissioned railway components can be effectively reused in alternative applications, as investigated by Sanudo et al. [2]. In particular, recycled concrete from sleepers can be employed as inert aggregate, reducing the overall carbon footprint. Steel components can be downsized and reused as secondary raw materials or reintroduced into railway applications, significantly lowering environmental impacts associated with primary steel production. In this context, Potapov et al. [3] investigated the feasibility of reusing deteriorated rails in underground metro systems as a strategy to extend their service life.

In parallel, increasing attention has been devoted to the recycling of polymeric components such as HDPE and nylon, which exhibit high durability and valuable mechanical properties. Several studies have investigated the use of recycled plastics in railway-related applications and composite materials. As an example, Dolci et al. [4] evaluated the use of recycled plastics in the production of the railway sleeper outer shell, with the aim of mitigating the related environmental impact. Zhang et al. [5] investigated the production of these components using composite materials based on recycled polyolefins (e.g., high-density polyethylene and polypropylene) reinforced with glass fibers, owing to their relatively high flexural performance.

Polyethylene, which accounts for a major share of global plastic production, is particularly suitable for multiple recycling cycles and represents a key resource for mitigating plastic waste impacts [6]. Mechanical recycling, compared to landfill disposal and incineration, leads to significant energy savings and reduced emissions of greenhouse gases and harmful substances [7]. This approach also contributes to decreasing the demand for virgin materials, resulting in additional environmental benefits.

However, recycled polymers often exhibit some degradation in mechanical properties due to microstructural inhomogeneities caused by contaminants or fillers [8]. This limitation can be mitigated by blending recycled polymers with virgin materials. For example, Zhang et al. [9] showed that the incorporation of virgin HDPE improves tensile and fatigue performance by reducing crack propagation rates. However, recycled materials, that exhibit significantly reduced properties in comparison to their virgin counterparts, are less attractive for widespread applications, thereby undermining their market potential. Similarly, the improvement of their performance through the addition of virgin material can be economically disadvantageous, reducing the overall benefits associated with recycling.

Alternatively, mechanical performance can be enhanced through reinforcement strategies involving fibers or fillers. Spychała et al. [10] demonstrated that adding ground glass fiber-reinforced plastics (GFRPs) to recycled HDPE increases stiffness, although it may slightly reduce tensile strength due to increased porosity. The incorporation of recycled or natural reinforcements, such as cellulose fibers, has also been shown to improve mechanical and thermal properties [11]. More advanced approaches involve hybrid reinforcement systems. For instance, the combined use of aluminum powder waste and carbon fibers in recycled HDPE matrices leads to synergistic improvements in mechanical properties and thermal stability [12]. In general, reinforcing recycled polymers with high-performance fibers broadens their application range and enhances their value.

In general, the reinforcement of recycled plastics with high-performance fibers represents an effective approach to increase mechanical strength and stiffness, also resulting in broadening the range of potential applications and enhancing the overall value of materials derived from waste streams. In particular, the incorporation of both virgin and recycled carbon fibers offers a promising and sustainable strategy to extend the use of recycled materials, while reducing the consumption of virgin resources.

In this context, carbon-based reinforcements, including carbon fibers and graphene, have attracted considerable interest for improving mechanical, electrical, and functional properties of polyethylene-based materials [13,14]. Borkar et al. [13] demonstrated that the addition of waste carbon to recycled HDPE significantly enhances tensile and flexural properties, enabling processing via injection molding and additive manufacturing. Carbon fibers are widely used due to their exceptional strength-to-weight ratio, high thermal and electrical conductivity, low thermal expansion and abrasion coefficient, and dimensional stability [15,16,17].

However, their production is highly energy-intensive—up to 595 MJ/kg, approximately 14 times higher than steel production—resulting in significant environmental impacts [18,19]. Therefore, recycling carbon fibers is essential to reduce both environmental burdens and material costs. Recycled carbon fibers, although generally exhibiting lower properties than virgin fibers, retain sufficient performance for many industrial and civil engineering applications [20]. Among available recycling methods, mechanical recycling is particularly attractive due to its low cost and reduced environmental impact. The cost and energy consumption associated with recycled carbon fibers can be as low as 15% of that of virgin fibers, even in less favorable scenarios [21].

In this study, two types of carbon fiber waste are considered: dry fiber scraps from production processes and recycled fibers from composite materials, often containing residual matrix and impurities. Melt compounding via twin-screw extrusion is widely used to process recycled HDPE and carbon fibers, ensuring homogeneous dispersion of reinforcements within the polymer matrix [22]. Fibers derived from dry waste streams often retain their original sizing, facilitating interfacial bonding, whereas fibers obtained through processes such as pyrolysis typically require additional surface treatments [23,24,25]. Contaminants can negatively affect composite performance, as shown by Al Zhami et al. [24].

In this work, recycled HDPE bushes obtained from railway sleeper recycling processes are investigated. These materials are highly heterogeneous and contaminated with concrete particles, oxides, and other debris. Additionally, recycled carbon fibers in the form of non-woven textiles and fiber bundles are used. Both material streams originate from environmentally sustainable recycling processes.

A life cycle assessment (LCA) was conducted using both primary and averaged data to evaluate environmental performance. Recycled carbon fibers were compared with virgin counterparts, while selected polymer compounds were benchmarked against both virgin and average recycled HDPE. Extensive material characterization was performed. Carbon fibers were analyzed in terms of size distribution and purity (via TGA), while recycled HDPE was characterized for thermal stability (TGA), thermal properties (DSC), and melt flow behavior. High viscosity observed during preliminary testing required the addition of a slipping agent to facilitate processing. Several polymer blends and HDPE/carbon fiber composites were developed and characterized in terms of thermal, mechanical, rheological, morphological, and electrical properties. Interfacial adhesion between HDPE and carbon fibers is known to be a critical limitation; thus, maleic-anhydride-grafted polyethylene was initially used as a coupling agent [26].

However, alternative strategies were also explored. In particular, recycled polyethylene derived from post-consumer PET bottle closures was investigated as a sustainable alternative to conventional coupling agents. These materials, although not suitable for closed-loop recycling in food-contact applications under European Food Safety Authority (EFSA) regulations, are promising for open-loop recycling scenarios. A worst-case scenario was considered, involving mixed-color post-consumer closures. These recycled polymers were compounded with HDPE from railway bushes and processed with the slipping agent. Composites reinforced with recycled carbon fibers were also produced and subjected to the same characterization protocol.

The novelty of this work lies in the combined and systematic use of highly heterogeneous recycled HDPE streams—originating both from railway sleeper components and post-consumer closures—together with recycled carbon fibers obtained from multiple recovery routes. Unlike previous studies, this work explores alternative strategies to conventional coupling agents by employing recycled polymeric additives, aiming to simultaneously enhance interfacial adhesion and overall sustainability. Furthermore, the study integrates a comprehensive experimental characterization with an environmental assessment through LCA, providing a holistic evaluation of both material performance and environmental impact. This approach enables the identification of viable pathways for the valorization of complex waste streams into high-performance, sustainable composite materials.

The manuscript is structured as follows: Section 2 describes materials and experimental methods; Section 3 presents and discusses results, including material properties and environmental assessment; and Section 4 summarizes the main conclusions and outlines future perspectives.

## 2. Materials and Methods

Dirty HDPE bushes were supplied by Calcestruzzi Cipiccia SPA (Terni, Italy), which is a company working on the disposal of concrete-based sleepers. These components are progressively reduced in volume through several milling stages. The bushes are collected at the early stages and are characterized by both fine residues, mainly deposited on their external surfaces, and coarse concrete residues, mainly trapped in their inner (hollow) regions.

Strips of scraped/waste TNT made of (post-industrial) recycled carbon fibers, as well as fibers of quite heterogeneous sizes, collected from the filtration plant, were provided by Carbon Task Srl (Biella, Italy). These materials represent by-products obtained during the carbon fiber recycling (down-cycling) process.

The slipping agent erucamide (cis-13-docosenoamide) Armoslip E, purchased from Bromochim (Milan, Italy), was used, while Fusabond MB256D, purchased from Dow (Milan, Italy), was used as a coupling agent. Finally, another kind of recycled polyethylene was obtained from PET bottle closure milling, made in various colors, to represent the worst scenario in terms of material inhomogeneity.

The HDPE bushes were stored directly at the plant used for the railway crossbeam work and were processed as received. For life cycle assessment purposes, transportation was also included in the supply chain.

A laboratory-scale process was developed, which can be easily scaled up to an industrial level, with a lower energy consumption compared to the lab-scale procedures.

Five batches of bushes were weighed using a kitchen balance with a sensitivity of 0.10 g. Their average weight was 616.2 ± 17.7 g. The material was then subjected to a first step of dry cleaning using compressed air provided through a closed-loop silicon pipe, featuring three circumferential holes (each one positioned at 120°) of 2.00 mm in diameter. The distance between these positions was 50 mm and the pipe total length was 1000 mm. This pipe was closed within a box together with the bushes and the process took approximately 75 s. The scraped concrete waste about (0.41 ± 0.07)%_Wt_ was collected and disposed of (landfill was assumed).

The cleaned bushes underwent a milling stage by means of a polymer recycling apparatus (Tria Milling machine (Milan, Italy)), characterized by a power input of 12 kW and a production rate of 10 kg/h. Also in this case, a small material loss was observed (0.89 ± 0.13)%, mainly consisting of concrete powder.

The obtained beads (2.60 ± 0.05 kg) were subjected to a wet cleaning process using water and compressed air (gurgling medium). Due to the polymer’s lower density than water, the polyethylene beads floated on the water surface at the end of the treatment, whereas foreign material (e.g., iron oxides, concrete, etc.) sank. This stage lasted approximately 4 min and the beads were sieved through a 5 mm mesh under moderate vibration.

Subsequently, the wet material, containing 3.70 ± 0.13%_Wt_ moisture, was dried under vacuum and collected. The polluted water was subjected to microfiltration and made available for subsequent cycles.

**Figure 1:**
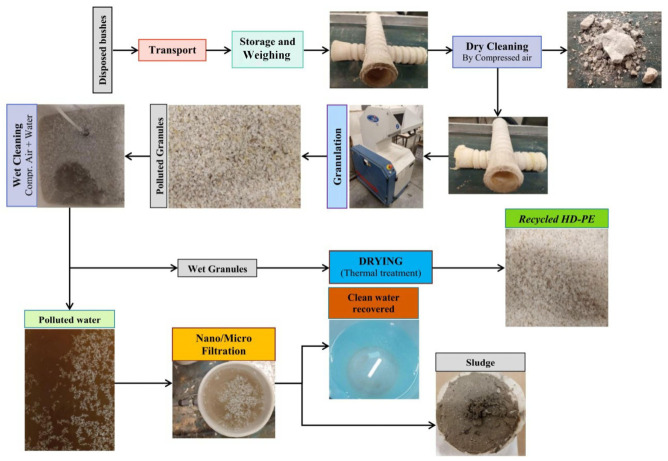
Developed lab-scale process used for the polyethylene recycling.

At the end of the process, the disposed water accounted for (4.95 ± 0.19)% in weight (the manual operation represents the worst scenario, because of a higher water loss in comparison to automated industrial processes), while additional concrete-based residues were disposed of (final sludge shown in Figure 1).

The same milling apparatus was used in the production of polyethylene beads from bottles closures, which were used in the final phase of the research activity.

**Figure 2:**
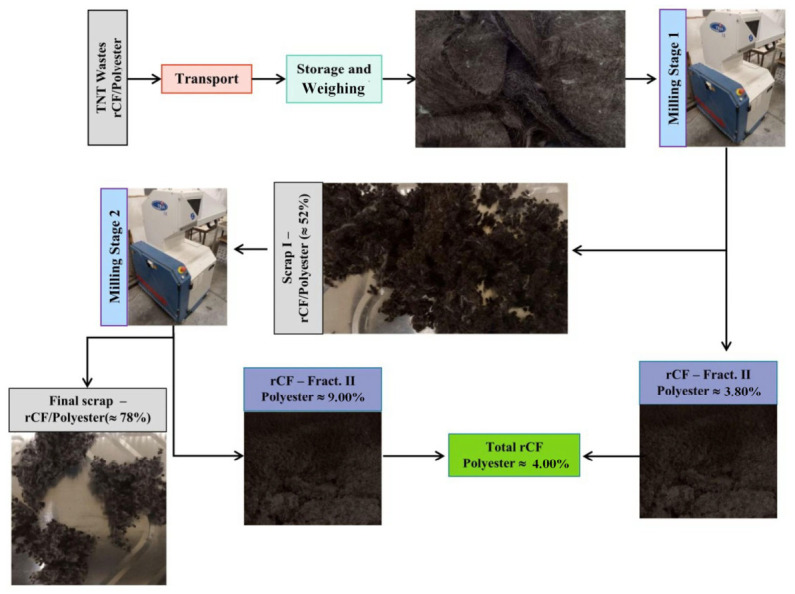
Developed lab-scale process used for the carbon fiber recycling.

For carbon fiber wastes, primary data regarding the related transportation were used (630 km in this case). The process scraps are characterized by 16% polyester fibers by weight, as originated from carbon fiber fabric remnants, scraped in the production of prepregs and dry fabrics (i.e., plain wave, twill, etc.) (Figure 2).

Batches made of 68 g strips and 8 g fiber bundles were weighed and subjected to multiple milling stages over a total period of 5 min (the abovementioned apparatus was used, characterized by the same energy consumption as the worst scenario [27]). The sieve diameter is 5 mm and polyester-rich bundles represented the process retentate. At the end of the process, the polyester content in the fiber bundles was 4%_Wt_, as determined by thermogravimetric analysis (TGA).

At the early stages of this work, the recycled HDPE beads (rHDPE) were directly processed using a DSM Microcompounder 15 (MP Strumenti, Milan, Italy), equipped with co-rotating twin screws and a recirculation system. A mixing time of 60 s was applied and the thermal profile resulted in a melt temperature of 184 °C. The rHDPE alone was found to be nearly unprocessable because of its very high viscosity. To improve the processability, a low-viscosity compatibilizer and a slipping agent were incorporated into the blend. Accordingly, three types of blend were produced, in addition to the neat rHDPE:

- rHDPE/Erc: rHDPE (95%_Wt_) + erucamide (5%_Wt_);
- rHDPE/MB256: rHDPE (80%_Wt_) + Fusabond (20%_Wt_).
- rHDPE/MB256/Erc: rHDPE (76%_Wt_) + Fusabond (19%_Wt_) + erucamide (5%_Wt_).

All the produced materials were processed through the abovementioned microcompounder using the same conditions as for the neat matrix.

Dog-bone-shaped (ISO 527 1BA) [28] and disk-shaped specimens (35 mm diameter and 3 mm thickness) were produced by injection molding, using a DSM Micro Injection Moulding Machine 10 cc (MP Strumenti, Milan Italy), under the following conditions:

- Injection and mold temperatures of 210 °C and 55 °C respectively;
- Injection/holding pressure of 12 bar;
- Holding time of 8 s.

Before the described processing stage, each material was dried at 50 °C overnight in a forced convection oven.

The three blends were also reinforced with 10%, 20% and 30% recycled carbon fibers by weight.

Due to the increase in viscosity of the reinforced systems, process parameters were adjusted: the extruder temperature profile was increased to achieve a melt temperature of 198 °C, the screw speed in the mixing was reduced to 50 rpm and the mold temperature increased to 60 °C during the injection molding phase.

Finally, blends of recycled high-density polyethylene from bushes and recycled polyethylene from bottle closures (caps) were produced, the latter as a replacement for the coupling agent, while the slip additive was retained at 5% in weight with respect to the baseline system.

Therefore, the baseline system, labeled as PE_Bushes_, was based on the rHDPE/Erc blend, whereas the material based on bottle closures was labeled PE_Cap_. For the sake of brevity, the acronym rHDPE/Erc is rHDPE in the new produced blends:

- rHDPE90/PE__Cap_10: PE_Bushes_ (90%)/PE_Cap_ (10%);
- rHDPE80/PE__Cap_20: PE_Bushes_ (80%)/PE_Cap_ (20%);
- rHDPE70/PE__Cap_30: PE_Bushes_ (70%)/PE_Cap_ (30%).

The system rHDPE80/PE__Cap_20 was then selected, after a complete characterization, to be reinforced with 10%, 20% and 30% recycled carbon fibers by weight.

The processing parameters for the unreinforced blends were identical to those applied for the production of the initial neat systems, both in the extrusion and in the injection molding phases. Similarly, the reinforced blends were processed under the same conditions as their corresponding counterparts based on the initial systems.

All the materials used in this work were subjected to a preliminary characterization. In particular, thermal and thermogravimetric analyses were performed on each individual component used for the blend production to determine the appropriate processing thermal window. Additionally, the melt flow index of the used matrices was evaluated to assess the related processing ability.

Thermal analysis was carried out on all the produced systems, composite materials included, by differential scanning calorimetry. A Mettler DSC, model DSC822A (Mettler Toledo, Milan, Italy), was employed according to the following experimental methodology:

- First and second heating scans from −50 °C to 250 °C at a heating rate of 10 °C/min. The first scan is used to erase the material previous thermal history;
- Cooling scan from 250 °C to −50 °C, at a cooling rate of 10 °C/min.

Thermogravimetric analysis (TGA) was performed on the recycled carbon fibers with the aim of investigating the residual polyester fiber content. It was also carried out on the produced blends and their composites. A thermogravimetric analyzer, Seiko, model TG/DTA6300 (TA Instruments, Newcastle, UK), was used, according to the following procedure:

- First heating scan from 30 °C to 600 °C, under an inert atmosphere (N_2_), at a heating rate of 20 °C/min;
- Second (cleaning) scan from 600 °C to 900 °C, under an oxidizing atmosphere (air), at a heating rate of 20 °C/min;

For both the DSC and the TGA characterization, three to five samples per system were tested to evaluate the results’ repeatability.

A preliminary optical characterization was carried out on the milled fiber obtained from the strips and on those collected from the filters. The former represented 90% of the used reinforcement, while the latter represented the remaining 10% in weight. This partition approximately reflects the actual distribution of wastes generated during the non-woven mat TNT production using the recycled fibers.

A reflection optical microscope, Nikon, model EPIPHOT 300 (Nikon Europe BV, Amstelveen, The Netherlands), was used with the aim at estimating the dimensions of the fibers.

As previously mentioned, the melt flow index of the initial materials and the produced blends was also investigated, using the MP Instruments Melt flow indexer (MP Strumenti, Milan, Italy). The test was conducted according to the standard ISO 1133 [29], at a temperature of 190 °C and under an applied load of 10 kg.

The obtained materials have been subjected to a mechanical characterization, using a Lloyd Instruments model 30K (Bognos Regin, UK) equipped with a 30 kN load cell. A tensile test was carried out at room temperature, in accordance with the ISO 527 standard [28]. The initial specimens’ gage length was 50 mm and an elongation rate of 5 mm/min was applied. At least seven samples per material were tested.

Electrical characterization was performed to determine the material surface and volume resistivity. A Keithley 8009 (Tektronix, Milan, Italy) was used, applying 8 alternate constant voltage (V^+^ to V^−^) electrification cycles, each one lasting 40 s. The applied voltage depended on the material conductivity; for example, a value of 500 V was used for insulating materials, while progressively lower voltages were used for dissipative and conductive materials (minimum applied value of 1 V).

Finally, a morphological characterization was carried out to investigate the composites fiber/matrix interface and the fiber alignment. A field emission scanning electron microscope (FE-SEM), Zeiss, model Supra 25 (Zeiss Italia, Rome, Italy), was used for this purpose.

## 3. Results and Discussion

### 3.1. Preliminary Results on Each Single Material Used in the Blends

Preliminary characterization was performed on each individual material used in the produced blends.

**Figure 3:**
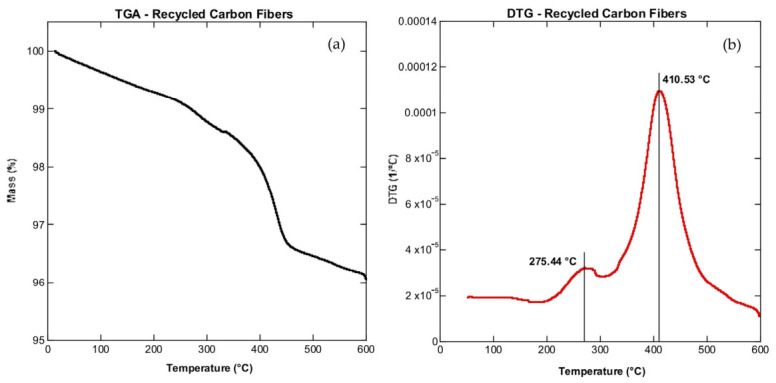
Weight loss (**a**) and DTG (**b**) for the recycled carbon fibers used as a reinforcement.

Regarding the TGA of the recycled fibers (Figure 3a), the weight loss at 600 °C was very low and a residual mass of 96.0% (±0.1%) was measured. The residue can be attributed to the carbon fibers, which, under a nitrogen atmosphere and below 600 °C, did not experience significant degradation [30]. This indicates that the milled fiber PET content was approximately 4% in weight. Because of this low polyester content, the reinforcement was mixed into the used matrices as obtained at the end of the milling stage, without additional energy-intensive treatment, aimed at increasing the purity, which is consistent with one of the objectives of the study.

Two main degradation mechanisms can be identified from the weight loss and the DTG diagrams (Figure 3b). The degradation occurring at the lower temperatures may be attributed to the residual epoxy [31] sizing on the fiber surface. Indeed, the low amplitude of the corresponding DTG peak indicated a very low amount of material, which is compatible with the typically low mass fraction of epoxy sizing on the carbon fiber used in the composite reinforcement. The related degradation mechanism occurred between 200 °C and 300 °C. Given the low amount, any additional degradation mechanism at the higher temperatures was hidden by the degradation of the other components, characterized by a considerably higher mass fraction.

The main degradation mechanism may be attributed to polyester fibers (3.98%_Wt_) included in the systems, as it appears compatible with the degradation of polyethylene-terephthalate-based polymers [32,33]. It occurred between 300 °C and 500 °C (peak at 410.5 ± 1.1 °C).

**Figure 4:**
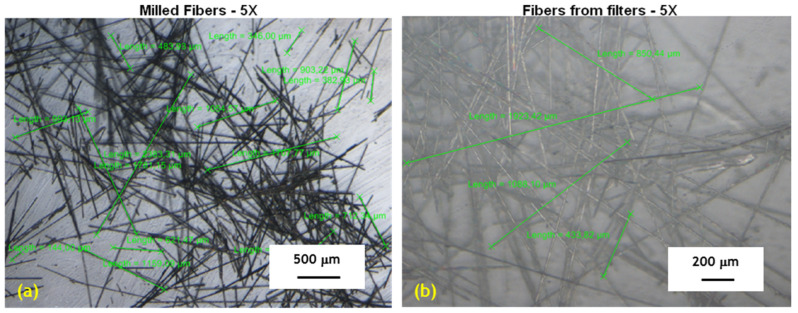
Optical image of the milled fibers (**a**) and those collected from the filters (**b**).

The optical analysis of the milled fibers (Figure 4a) and those collected from the filters (Figure 4b) revealed a broad distribution of fiber length, while the diameter showed a uniform distribution for both the materials.

**Figure 5:**
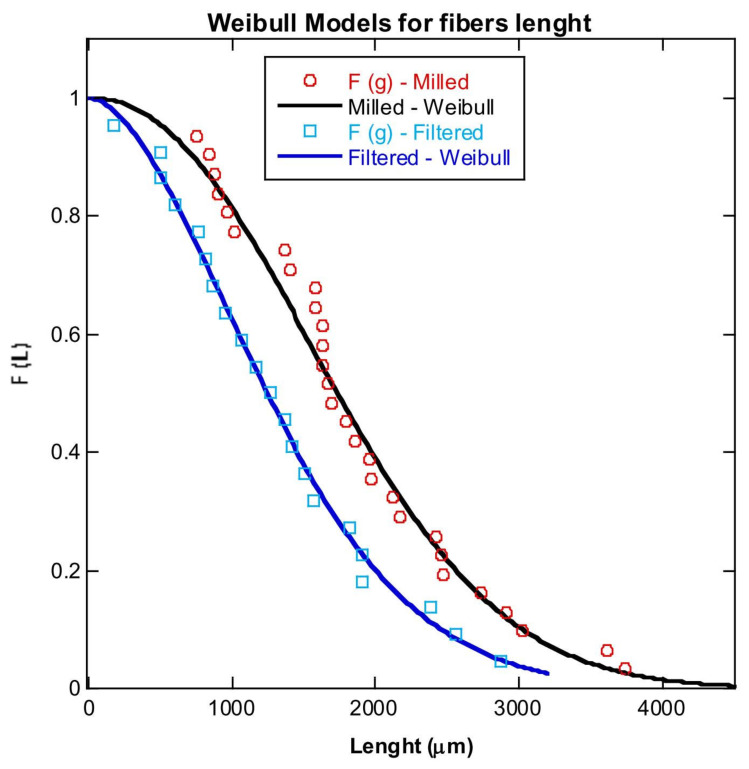
Weibull distribution of the investigated fibers length.

A statistical analysis based on the two parameters in a Weibull model (Equation (1)) was performed (Figure 5 and Table 1).

(1) $$F\left(x\right)=exp\left\{-{\left(\frac{x}{x_{0}}\right)}^{\alpha }\right\}$$

where x_0_ and α are the distribution scale parameter (in this case, the most probable value for the fiber length) and the shape factor, respectively.

For both types of reinforcement, the obtained length fell within the critical range [34] (1 ÷ 3 mm), suggesting a sub-optimal reinforcing (as well as stiffening) effect that is expected when the fibers are incorporated into the matrices.

**Figure 6:**
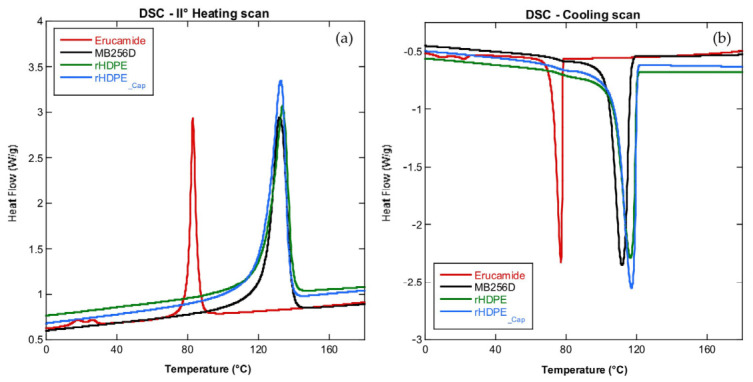
Neat matrices’ 2nd heating (**a**) and cooling scans (**b**).

Thermal analysis was carried out on the full range of materials used in the blends to determine their melting and crystallization temperatures. As summarized in Table 2, the melting temperatures (T_melt_) of the investigated recycled polyethylene, from the bushes and from the caps, fall within the typical range for high-density polyethylene (Figure 6a). The corresponding enthalpies (ΔH_melt_) were also very similar to those expected for this family of polymers [35]. The same can be claimed in terms of crystallization temperatures (T_cryst_) and enthalpies (ΔH_cryst_) for the two recycled materials (Figure 6b). The high value found in the case of the enthalpy indicates a high degree of crystallinity.

Thermograms of Fusabond indicated slightly lower melting and crystallization temperatures, as well as a slightly lower enthalpy. Additionally, a small peak at about 79 °C was observed which is characteristic of a maleate grafted polyethylene [36].

Erucamide had a lower value for the transition enthalpies and correspondingly lower transition temperatures.

**Figure 7:**
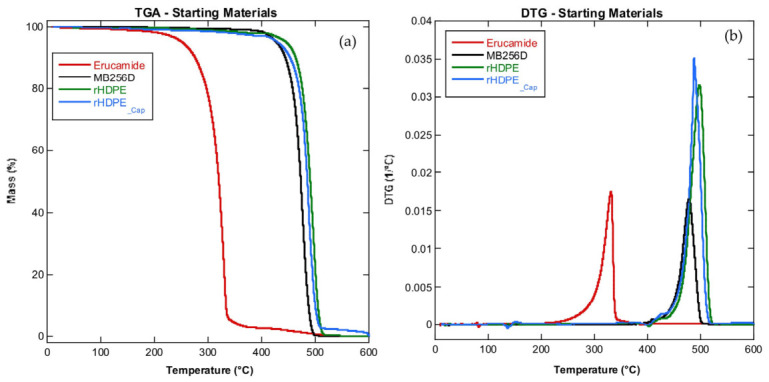
Neat matrices’ weight loss (**a**) and related DTG (**b**).

Despite being derived from post-consumer recycled materials, the polyethylene obtained from caps and bushes was characterized by the highest thermal stability (Figure 7a,b) among the investigated polymers. This was likely due to the intrinsic properties of these materials, including very high durability (low sensitivity to environmental aging) and a high resistance to thermal and mechanical degradation (Table 3). The polyethylene from bushes showed only a minor weight loss between 120 °C and 350 °C (lower than 3.50%), which can be attributed to traces of low molar mass chains or oxidation products formed during the related service life (mechanical loads and environmental weathering).

Fusabond also exhibited a high thermal stability, as it, similarly to the other PE-based polymers, did not experience any significant weight loss (<5%) up to approximately 378 °C. The degradation phenomenon ended at slightly over 500 °C (T_end_).

Erucamide was characterized by a considerably lower degradation temperature, as the weight loss started slightly beyond the threshold of 200 °C (onset degradation temperature T_onset_), followed by a quick decomposition, reaching the maximum rate at 331 °C (T_Max_).

### 3.2. Results Obtained for the Preliminary Blends and the Related Composites

The results previously shown in Section 3.1 were used as a guideline for choosing the most suitable processing temperatures for the realization of blends containing the slipping and coupling agents during the extrusion and the injection molding processes.

The produced blends were subjected to thermal, thermogravimetric, and mechanical characterization, as well as melt flow index (MFI) measurements. The MFI is very important for assessing processability and for estimating the expected behavior of composites during the extrusion and the injection molding phases.

As expected, the melt index of the recycled polyethylene was very low, and the addition of erucamide to the rHDPE alone was insufficient to improve the material flowability, as summarized in Table 4, despite this additive being generally considered effective in enhancing polyethylene processability.

The highest value of the melt index was obtained for the blends containing both the coupling and the slip agent, although the value of this property remained quite low. For this reason, some processing difficulties were observed, as expected, for the reinforced systems both in the extrusion and the injection molding phases. To mitigate these issues, the processing temperatures were slightly increased by only 5–10 °C both in the extrusion profiles and at the injection nozzle to avoid degradation phenomena, mainly for the blends containing erucamide.

In the case of composite production, the obtained blends’ high viscosity caused additional phenomena during the processing due to the high shear stresses occurring in the melt, including:

- Carbon fiber breakage;
- High degree of carbon fiber alignment along the flow direction during the injection molding process, resulting in a significant material anisotropy.

**Figure 8:**
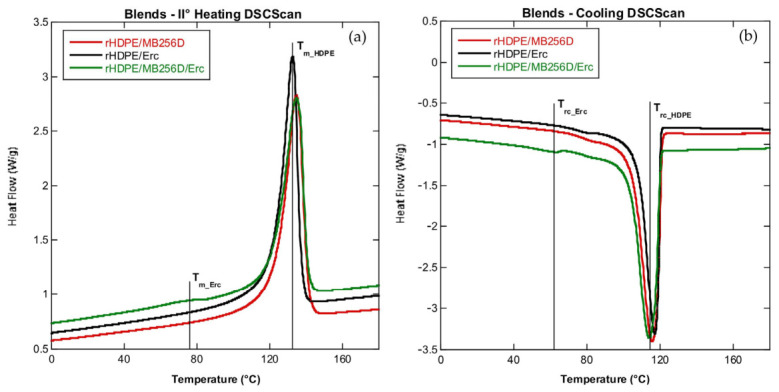
Blends’ 2nd heating (**a**) and cooling scans (**b**).

The thermal properties of the produced blends were generally similar to those of the starting material, as shown in Figure 8a,b. Blends containing 5% erucamide by weight were characterized by peaks with a very small amplitude corresponding to melt and recrystallization temperatures of this additive. Small differences can be attributed to experimental error.

It was also observed that erucamide caused a slight decrease in the blends’ melting temperature (Table 5), while crystallization temperature showed a more relevant decrease, likely due to partial degradation occurring during the extrusion process. The blends’ transition enthalpies remained within the same range as those of the neat matrices, indicating that the additional extrusion process did not lead to significant changes in the degree of crystallinity or widespread long-chain cleavage.

Carbon-fiber-reinforced systems did not show significant changes in thermal properties compared to the corresponding matrices (Figures S1 and S2 included in the Supplementary Materials).

**Figure 9:**
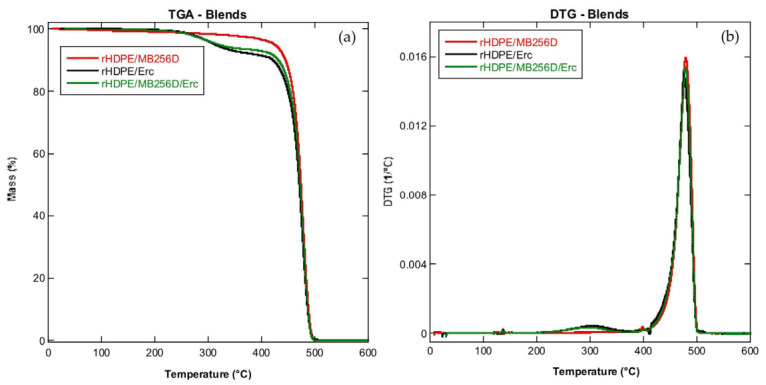
Weight loss (**a**) and DTG (**b**) for the produced blends.

Regarding degradation behavior, the rHDPE-based systems exhibited a slow, but continuous, weight loss from 100 °C to 350 °C (about 3.50%). This is likely due to traces of low molar mass chains or oxidation products formed during the bushes’ service life (environmental weathering), as shown in Figure 9a,b. The highest thermal stability was observed in the blends based on the rHDPE, which exceeded the stability of the Fusabond MB256D, probably because of the used polyethylene’s inherent stability, despite its previous service life and the applied recycling procedures. As previously shown, the erucamide additive exhibited the lowest thermal decomposition performance, which also negatively impacted on the performance of the other blends (data summarized in Table 6).

**Figure 10:**
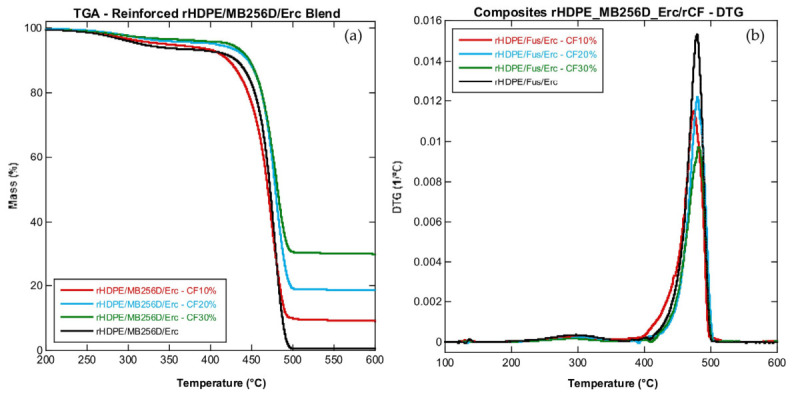
Weight loss (**a**) and DTG (**b**) of the reinforced blends.

As expected, the addition of carbon fibers to the matrices slightly decreased the systems’ weight loss rate and enhanced the related thermal stability. In the reinforced systems, both the weight loss and the degradation rate between 100 °C and 350 °C were lower than those of the corresponding unreinforced matrices (Figure 10a,b). This effect was likely due, in part, to the increased thermal conductivity of the reinforced systems, which facilitated faster dissipation of thermal energy and shifted the onset of the main degradation mechanisms to higher temperatures. For high-fiber mass fractions, an increase both in the onset temperatures and in the maximum degradation rate occurred, proportionally to the fiber mass fraction itself.

At the degradation onset temperature, HDPE underwent chain scission and formation of low-molecular-weight alkyl radicals. These species reacted with carbon fibers, which contributed to the described delay in the material degradation compared to the neat HDPE [37].

Neat matrices were characterized by a complete (100%) decomposition at the end of the test, whereas the composites retained a significant residue, which approximately corresponds to the mass fraction of the initial fiber content. Actually, the residue was slightly lower than the nominal mass fraction, because of polyester residues in the reinforcement, whose degradation mechanism did not significantly differ from the HDPE one. This observation indicates that polymer degradation is the prevailing mechanism and the related chain scission is the dominant decomposition reaction [37].

**Figure 11:**
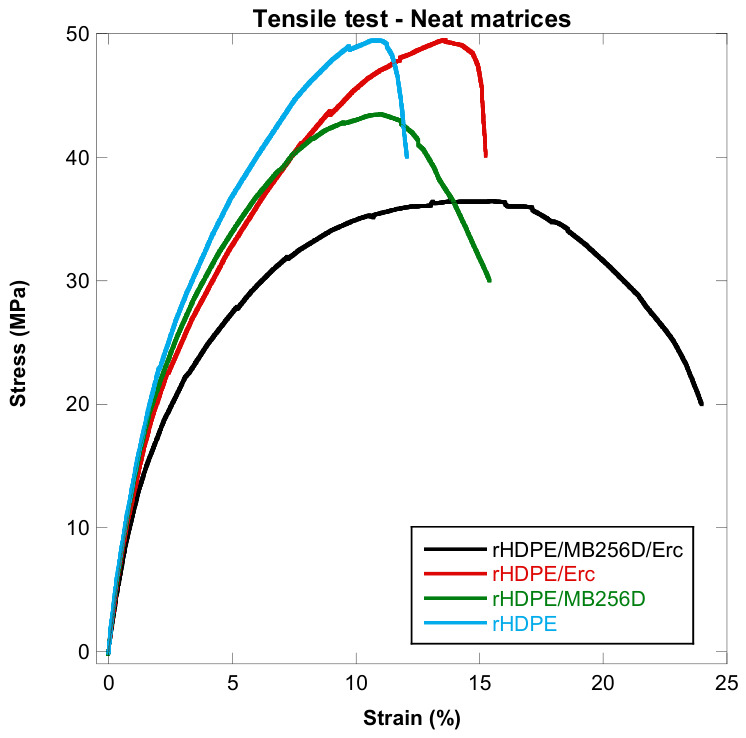
Tensile stress–strain curves for the unreinforced systems.

Mechanical characterization was performed for both the developed unreinforced and reinforced blends, as well as the recycled neat HDPE. Due to the neat polyethylene’s high viscosity and low processability, no reinforcement was incorporated, as many extrusion problems occurred. The resulting stress–strain curves obtained in the tensile test carried out for the neat matrices are shown in Figure 11.

The highest strength and stiffness can be attributed to the recycled polyethylene, which also exhibited the lowest strain. The addition of the coupling (compatibilizer) agent negligibly affected the blend mechanical properties, as the system resulted in a decrease in the yield strength (σ_Yeld_) and stiffness, as well as a slight increase in the yield strain and in the strain at break. The slip agent addition resulted in an increase in yield strain and strain at break (ε_Yeld_), accompanied by a decrease in the Young modulus (E). The system containing both the compatibilizer and the slip agent was characterized by the lowest stiffness and strength but also by the highest strain at break and yield strain.

**Figure 12:**
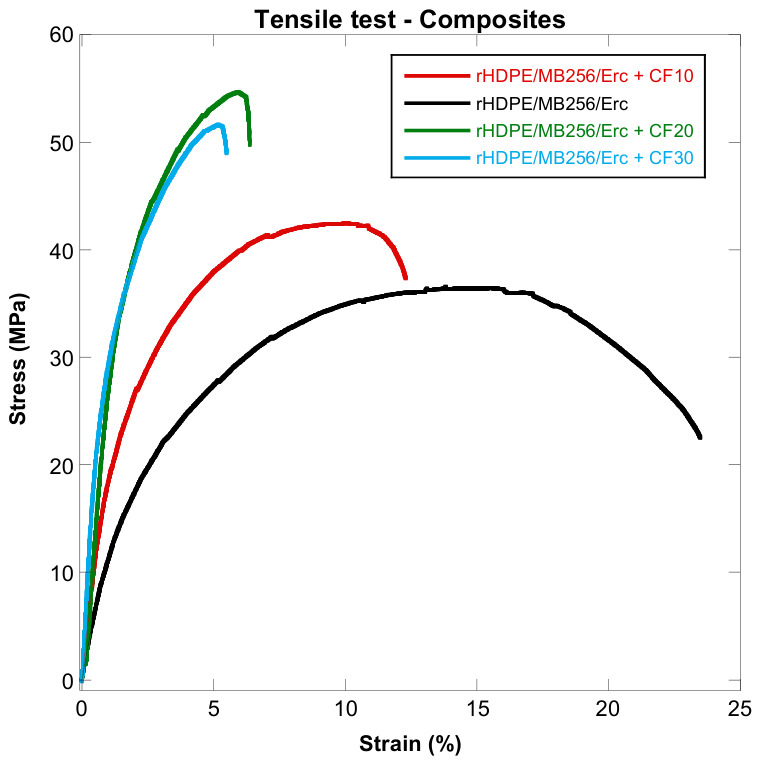
Tensile stress–strain curves for the reinforced systems containing recycled polyethylene, coupling and slip agents.

Figure 12 summarizes the results for one of the reinforced systems, specifically the blend containing the recycled polyethylene, the coupling and the slipping agents. It shows that fiber addition leads to a small improvement in the strength, with a maximum increase of 19% for the system containing 20% carbon fibers by weight. Conversely, fiber addition proportionally reduced the strain at break (up to 65%). Significant increases in the Young modulus (from 54% to 275%) were observed mainly for the grafted systems (Table 7). Additional information on the mechanical property trends of the blend and the corresponding reinforced systems is provided in Figure S3 in the Supplementary Materials.

An increase in the blend stiffness as the carbon fiber mass fraction increases is an expected outcome, as well as a decrease in the strain at break, due to the stiffening effects of the reinforcement. According to established models, these effects weakly depend on the fiber/matrix compatibility or interface, but they are strongly affected by the fiber volume fraction, fiber orientation with respect to the loading direction, and fiber length [38]. Also, the small increase in the systems’ strength can be considered an expected result, as it strongly depends on the fiber length, fiber/matrix interface and compatibility, as well as fiber alignment in comparison to the loading direction and the fiber mass fraction.

**Figure 13:**
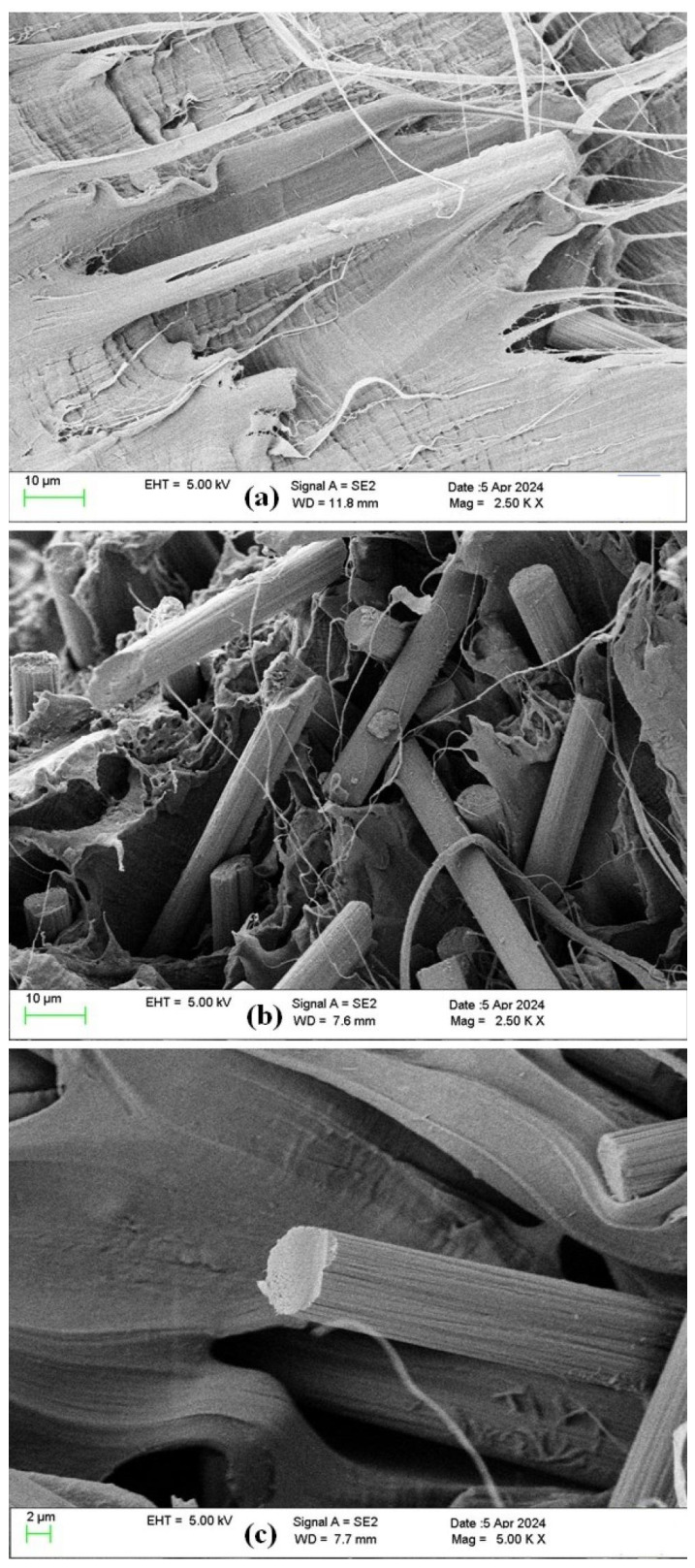
SEM regarding the blend HDPE/erucamide, reinforced with 10% (**a**), 20% (**b**) and 30% (**c**) recycled carbon fibers.

Morphological (SEM) analysis provided a partial explanation for the observed mechanical performance. As shown in Figure 13, for the blends based on recycled polyethylene and erucamide, a good interface was obtained in the system reinforced with 10% carbon fibers (Figure 13a). The fibers are fully embedded in the matrix, with few broken fibers rising from the surface and minimal pull-out phenomena. In contrast, higher fiber mass fractions led to a poorer interface, with widespread fiber pull-out and a large number of fractured fibers protruding from the surface (Figure 13b). These fibers displayed matrix residues over their surface, indicating a quite weak fiber/matrix interface. The system containing the highest load of carbon fibers showed a reinforcement with no traces of matrix on the fiber surface, indicating a lack fiber/matrix interaction (Figure 13c).

These outcomes explain the limited increase in the system tensile strength at the highest fiber contents and the significant decrease in the related strain at break. Actually, the low strengthening effects can also be explained by the small fraction of fibers exceeding the critical length.

**Figure 14:**
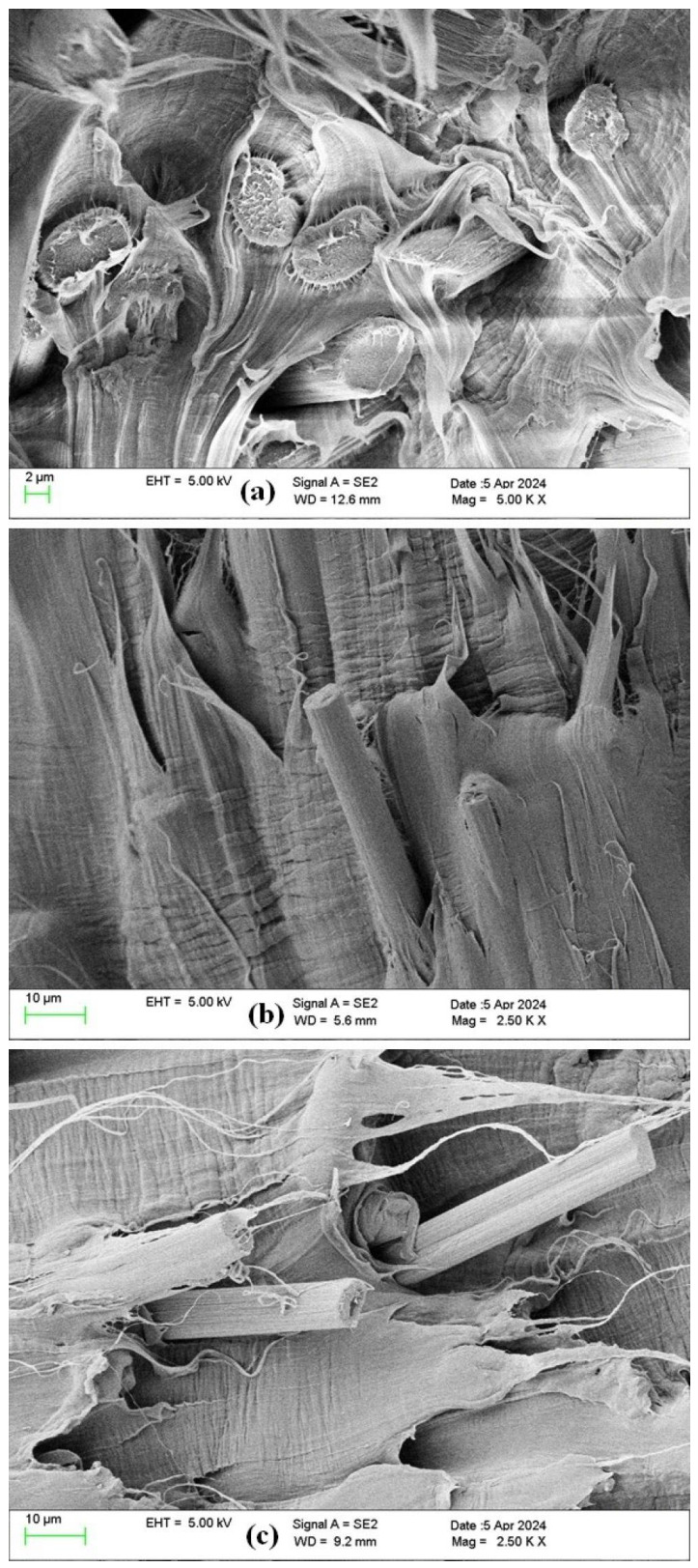
SEM regarding the blend HDPE/Fusabond MB256D, reinforced with 10% (**a**), 20% (**b**) and 30% (**c**) recycled carbon fibers.

Conversely, as shown in Figure 14a, the system made of the mix of recycled polyethylene and the coupling agent showed a strong fiber/matrix interface for the full range of fiber loadings. Few fibers rise from the fractured surface and most are completely embedded in the matrix (Figure 14b). No relevant fiber pull-out can be appreciated in the SEM images (Figure 14c).

These observations are fully consistent with the experienced mechanical performance, as it is the system characterized by the highest strengthening effect resulting from the reinforcement addition.

**Figure 15:**
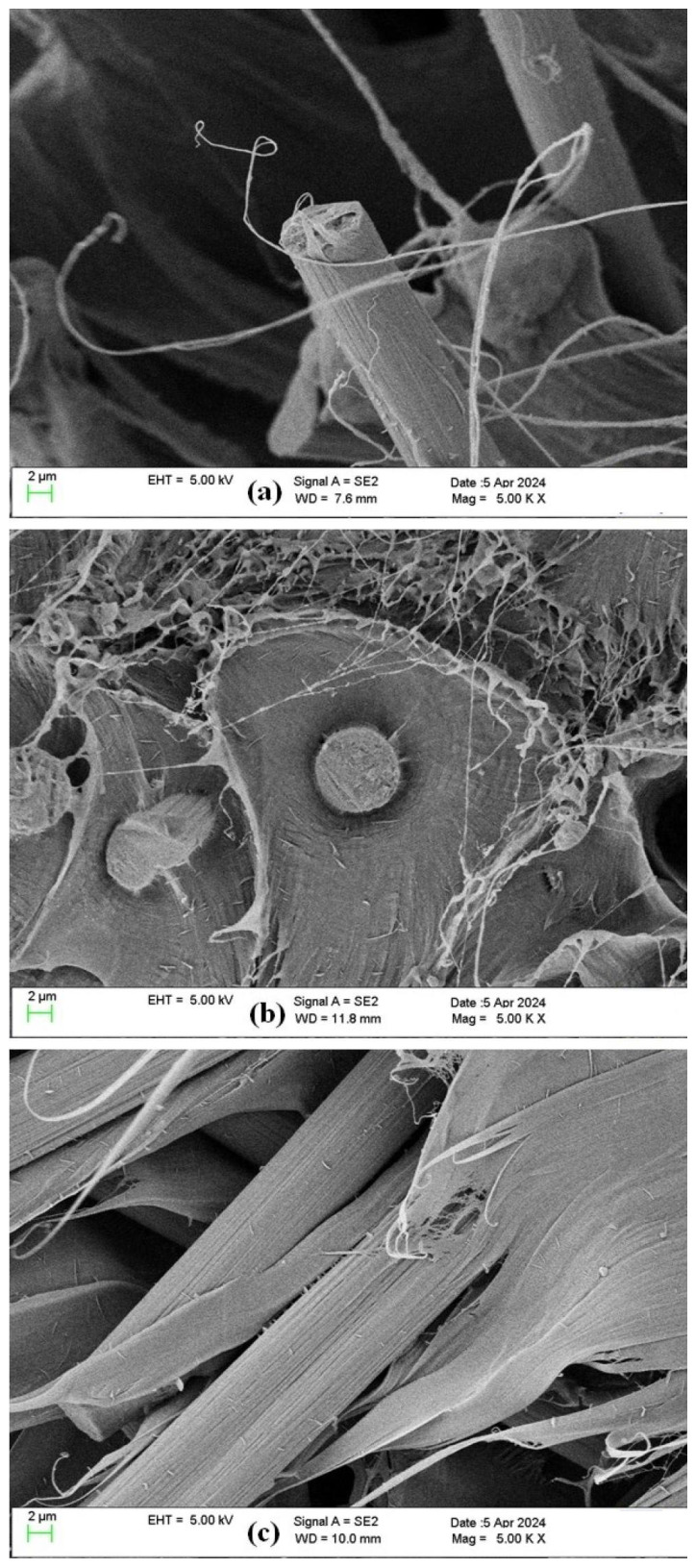
SEM regarding the blend HDPE/Fusabond MB256D/Erucamide, reinforced with 10% (Part (**a**)), 20% (Part (**b**)) and 30% (Part (**c**)) recycled carbon fibers.

Also, the system based on polyethylene, compatibilizer and the slipping agents showed a very good fiber/matrix interface, up to 20% carbon fibers by weight (Figure 15a,b), although some evidence of fiber pull-out can be found. In the case of the system reinforced with 30% carbon fibers, the reinforcement appears only partially covered by matrix residues, indicating an interface deterioration, likely due to an excess of fiber content, which prevents an effective wetting by the matrix. Across the full range of the reinforced systems, mainly at the highest fiber loading (20% and 30% in weight) was a significant fiber alignment along the flow direction observed (Figure 15c), which is characteristic of the injection molded specimens (Figure S4 in the Supplementary Materials). This was due to the very high matrix viscosity, which increased with the fiber content. This can be considered the main reason for the measured high stiffening effect of the reinforcement.

**Figure 16:**
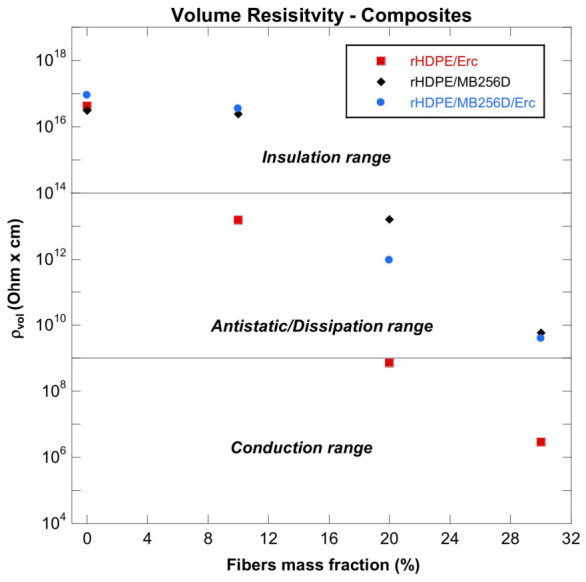
Volume resistivity of the produced composites.

Fiber parallelism did not significantly hinder their mutual contact, even at the highest mass fractions. However, this contact did not directly lead to mechanical stress transfer among the fibers, which probably occurs only in compatibilizer-rich systems. Conversely, an incompletely covered reinforcement, characterized by a sub-optimal fiber/matrix interface, can show high electrical conductivity, as a minimal barrier to the charge pathways is provided. In contrast, a strong fiber/matrix interface can significantly hinder the charge transfer between the fibers. This behavior explains the results obtained for the developed composites in terms of electric conductivity. In general, the addition of carbon filler and fibers enhanced the matrix electrical conductivity proportionally to the related mass fraction, as shown in Figure 16 and Table 8, and lowered the percolation threshold [38].

Although the fiber or the reinforcement aspect ratio significantly affects the percolation threshold, in general, the most common value for HDPE/carbon fiber composites ranges between 12% and 16% in weight [39], as confirmed by the measurements for all the systems developed in this investigation.

Materials based on the neat matrices or reinforced with 10% carbon fiber exhibited a low conductivity, confirming that below the percolation threshold every system behaves as an electrical insulator. In this regard, the order of magnitude of the volume resistivity (ρ_vol_) exceeded the range of 10^12^ ohm * cm, as shown in Figure 16 (Figure S5 in the Supplementary Materials deals with the trend of the surface resistivity (ρ_sur_) versus fiber mass fraction for this material).

The composites based on polyethylene and compatibilizer behaved as insulating materials, even at the highest fiber mass fraction, due to the strong fiber/matrix interface. These systems are potentially suitable for applications requiring good toughness combined with electrical insulation. The system based on HDPE and erucamide showed dissipation properties at 20% reinforcement by weight and a moderate conductivity at the highest fiber load due to the high reinforcement content and the poor fiber/matrix interface. This group of materials is potentially suitable for applications requiring high stiffness and electrical conductivity. Their drawback is the related brittleness.

Finally, reinforced systems based on polyethylene, compatibilizer, and slipping agents performed with an intermediate electric resistivity. Even above 20% carbon reinforcement by weight, these materials did not significantly exceed the dissipation/conduction limit. Their properties represent a good compromise in terms of stiffness, strength, and conductivity, although the strain at break remains very low.

### 3.3. Results Obtained for the Further Blends and the Related Composites

As discussed in the previous sections, the whole range of the developed composites showed high mechanical stiffness and brittleness (low strain at break), mainly in the case of the systems containing a low amount of compatibilizer. On the contrary, the composites based on the compatibilized blends were characterized by a slightly reduced brittleness and increased strength but very low electrical conductivity.

To address these features, additional systems were developed with the aim of producing materials characterized by fully tunable properties and low potential environmental impact. In this regard, the coupling agent was replaced with recycled polyethylene from bottle closures, with the goal of increasing the material strain at break while maintaining electrical conductivity. This was achieved through the formation of separated phase systems, where the interphase regions could act as preferential locations for the reinforcement. This theoretically offers two advantages:

- Slight deviation of the fibers from the flow direction, which potentially increases the contact points among them, increasing the system conductivity;
- Increased amount of amorphous interphase regions enhancing the system deformation under applied loads.

The first benefit obtained through the addition of recycled polyethylene from bottle closures was a significant increase in the melt index (this trend is shown in Figure S6 in the Supplementary Materials), as summarized in Table 9. Even the blend containing only 10% polyethylene by weight from the closures showed a higher melt index than the rHDPE/MB256D/Erc system. This property increased as the weight fraction of the recycled polyethylene increased, following a linear trend. This addition also improved the system processability.

**Figure 17:**
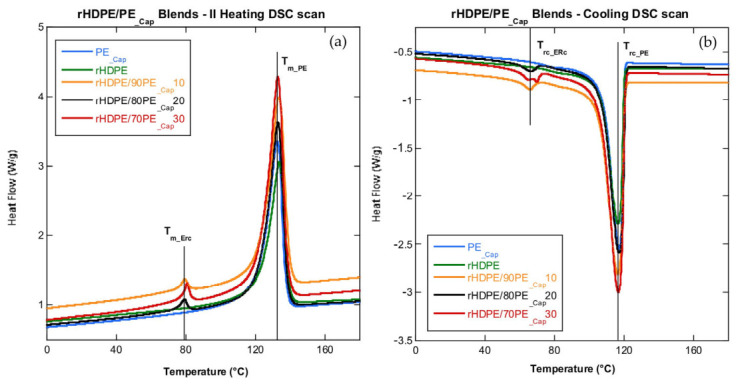
Blends of the two recycled materials—2nd heating (**a**) and cooling (**b**) scans.

Figure 17 shows the second heating (a) and cooling (b) scans of the investigated bends, while Table 10 summarizes the measured thermal properties.

The baseline system was made of recycled polyethylene (high density) from the bushes and erucamide as a slip agent. The presence of erucamide is also confirmed by the DSC thermograms of the blends, as the related melting and crystallization peaks were clearly detected. Additionally, the melting and the crystallization peaks of the main component (rHDPE) were observed. On the contrary, the DSC thermogram regarding the PE recycled from the bottle closures only exhibited the crystallization and the melting peaks of the neat matrix.

The thermal properties of the blends were not significantly affected by the inclusion of the other kind of polyethylene. As expected, carbon-fiber-reinforced systems did not show significant changes in the thermal properties in comparison to the corresponding matrices (Figures S7 and S8 in the Supplementary Materials).

**Figure 18:**
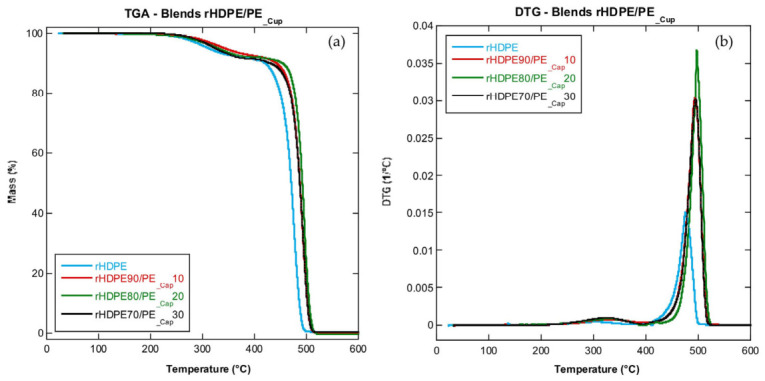
Weight loss (**a**) and DTG (**b**) of the produced PE_Bushes_/PE_Cap_ blends.

The addition of the recycled polyethylene from bottle closures also increased the blends’ thermal stability in comparison to the baseline material, as shown in Figure 18 (system containing rHDPE sourced from the bushes and erucamide). This improvement was primarily attributed to a lower level of degradation of the polyethylene recycled from the caps in comparison to that obtained from the bushes. Even if the former material has been recycled from post-consumer components, its prior service life is characterized by lower environmental and mechanical stresses than the latter one, thus having a reduced weathering level. Furthermore, the observed results (Table 11) may also reflect the inherently higher thermal stability of the material used in the closure compared to that used in the bush production.

The best performance was observed in the system containing 20% polyethylene by weight from closures. For this reason, it has been selected for the production of the composites reinforced with the recycled carbon fibers. The resulting composites showed a thermal stability similar to that of the corresponding matrix (Figure S9 and Table S1 in the Supplementary Materials), with only a minimal increase in the onset degradation temperature.

**Figure 19:**
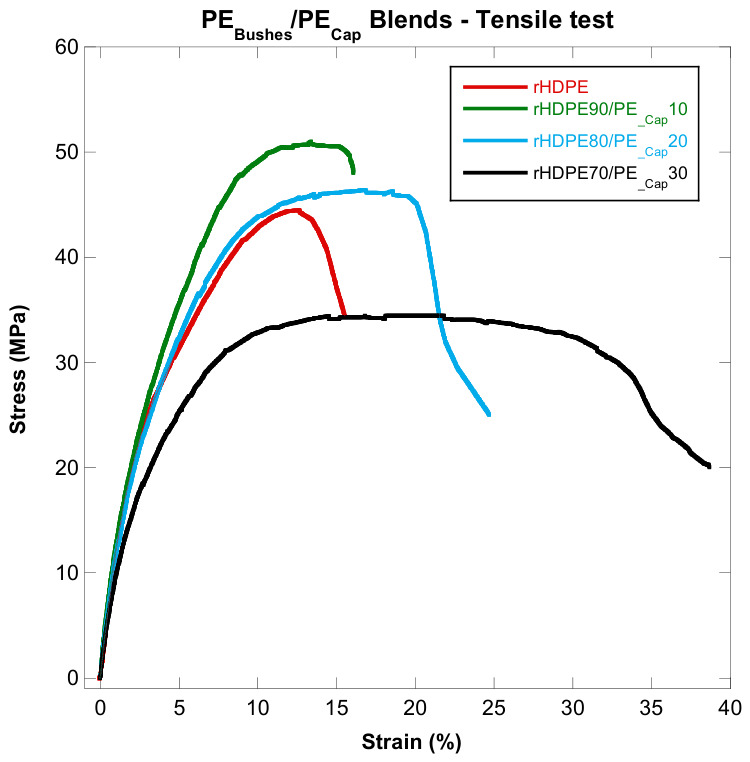
Tensile stress–strain curves for the unreinforced systems based on the two different kinds of polyethylene.

Regarding mechanical characterization, the addition of the polyethylene from the closures did not lead to an increase in the tensile strength and stiffness. In fact, as the mass fraction of this component increased, the blends’ Young modulus (stiffness) and strength decreased. On the contrary, the strain at break is proportionally increased with content of polyethylene from the bottle closures (Figure 19).

**Figure 20:**
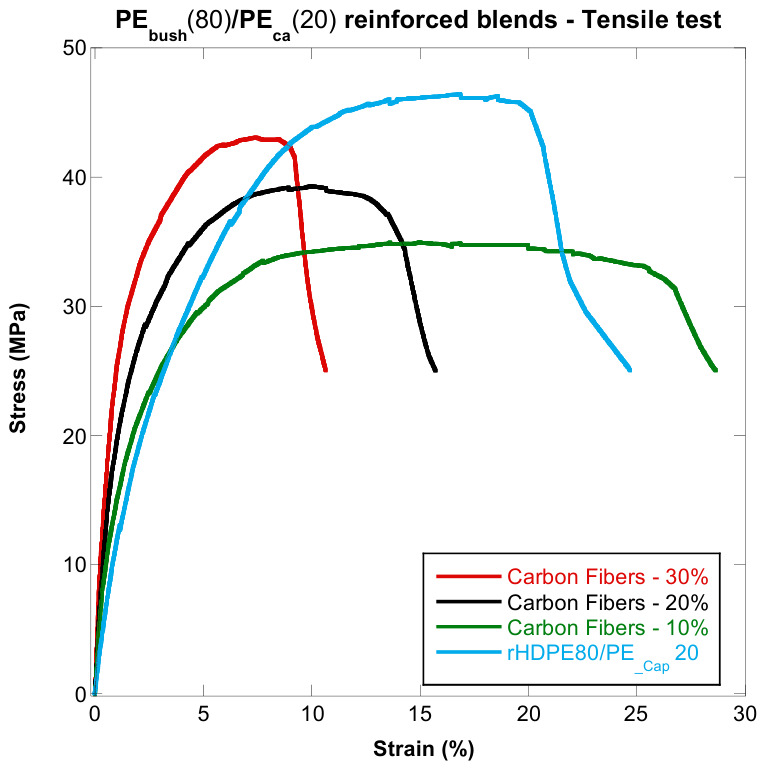
Tensile stress–strain curves for one of the reinforced PE_Bushes_/PE_Cap_-based systems.

**Figure 21:**
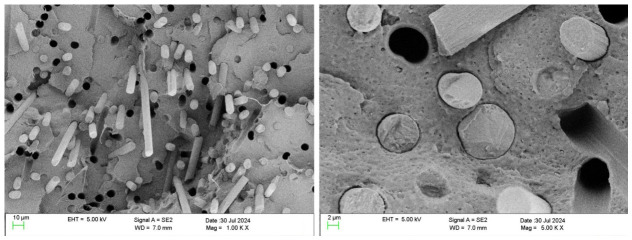
SEM of the blend PE_Bushes_/PE_Cap_, reinforced with carbon fibers (10% in weight).

The system containing 20% PE by weight from caps represented the best compromise in terms of thermal, rheological, and mechanical properties, as summarized in Table 12. As shown in Figure 20 and in Table 13, the system reinforced with 10% carbon fiber by weight exhibited the lowest performance and homogeneity. Similarly to the other systems, the composites’ Young modulus increased as the fibers’ weight fraction also increased. The reinforced blends showed a lower tensile strength and strain at break compared to the neat matrix of the blends previously dealt with. This behavior was attributed to the increased heterogeneity and a poorer fiber/matrix interface, as illustrated in Figure 21.

The morphological analysis evidenced a poorer fiber/matrix interface in comparison to the compatibilized systems, as well as several fiber pull-out events, even at the lowest reinforcement mass fractions. Also, in this case, the fibers appeared aligned along the flow direction, but they were in relatively close contact, which promotes the formation of a conduction pathway.

For these systems (Table 14) the percolation threshold occurs between 12% and 16% carbon fiber by weight. At low (10%) fiber mass fractions the composite behaved as an insulator, while at mass fractions equal to or greater than 20%, the system was characterized by a dissipative or a weakly conductive behavior.

In comparison to the previously developed systems, these blends were only characterized by an increase in the strain at break, resulting from a higher heterogeneity. No improvements in the electrical conductivity were observed. Nonetheless, the systems represent an alternative to the blends containing the compatibilizer, in case a higher conductivity is desired in a less brittle material.

### 3.4. Process Sustainability Investigation by Mean of LCA

The main scope of the whole work was the development of a highly scalable, cost-effective, and environmentally sustainable mechanical recycling methodology, along with the production of marketable materials, characterized by a satisfactory performance and tunable properties.

A low environmental impact was considered the key prerequisite throughout this investigation and was assessed by means of a preliminary life cycle analysis (LCA).

The analysis was conducted across the entire supply chain of the materials used in the production of the baseline blends and the related composites investigated in the study:

- System based on a mixture of recycled polyethylene derived from the bushes (95% in weight) and erucamide (5% in weight);
- Recycled carbon fibers.

The LCA was developed in accordance with the ISO 14040:2006 [40] and ISO 14044:2006 [41] standards.

The adopted approach was consistent with the goal and scope of this study, as it is essential to effectively assess the environmental sustainability of the developed materials, through the LCA methodology.

In brief, the goal of the analysis concerns the assessment of the environmental impact of each material and the related recycling methodology and providing a preliminary comparison with the virgin counterparts and baseline recycled systems included in the used software database.

It should be noted that this comparison represents only a preliminary and approximate verification of the sustainability of the produced material. Indeed, the reference datasets included in the software database are based on averaged and validated data. On the contrary, the models developed for the recycled materials produced in this study rely on direct measurements and/or calculations and estimations. The latter mainly concern data related to the energy intensity of processes at an industrial scale.

This analysis enables the identification of potential environmental hotspots within the life cycle (on the basis of a cradle to gate approach) of the investigated materials, with particular attention on the most critical processes and materials from an environmental perspective.

Parametric models have been developed to allow continuous updates in parallel with ongoing technical advancements, both within and beyond the timeframe of the present study.

As previously outlined, this approach was applied to all the processing technologies, materials, and ancillary components whose models were not available in the software libraries or databases (Ecoinvent 3.12.).

A “cradle to gate” approach has been applied, with no issues related to transportation towards the end user, service life and end of life scenarios.

The life cycle analysis was carried out by means of the software SimaPrò v 10.3.0 and the life cycle impact assessment (LCIA) was performed by means of the tool ReCiPe 2016 (considering both mid-term impact categories and end-point indicators).

The amount of 1 kg was considered as a functional unit for all the investigated (recycled) products.

Regarding the system boundaries, for each manufacturing stage, the corresponding technological/physical boundaries have been taken into account. Materials, energies, emissions and wastes crossing the system during the whole range of operations, and actually carried in the recycling routes for the polyethylene and the carbon fibers, have been included in the system boundaries.

The topics of data quality, assumptions, limitations, etc. were addressed in the inventory phase and separately dealt with case by case for each system under investigation. It should be noted that, for input materials—including scraps and waste, as well as for transport—primary data were employed. The energy consumed during milling, as well as during dry and wet cleaning stages, was calculated through the nominal power rating of the used devices, without applying any reduction factors, and assuming the maximum observed processing times. This represents a worst-case scenario, as pilot-scale equipment characterized by low production rates and higher waste generation was employed. Scaling up the process is expected to reduce energy consumption and improve environmental performance.

**Figure 22:**
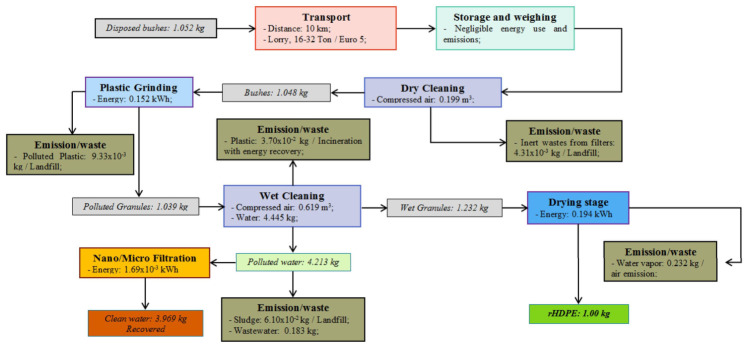
Flow chart regarding the production of 1.00 kg of a baseline recycled polyethylene compound.

The procedure used to produce the recycled polyethylene beads has already been described in the Section 2. Ninety-five percent of this material by weight was compounded via extrusion with 5% erucamide, as shown in the flow chart of Figure 22.

The material, collected from the railway crossbeam recycling facility, is transported to the polyethylene recycling plant by means of a 16–32-ton lorry (Euro 5). The real distance was considered, which amounts to 10 km. After the storage and weighing operations, assumed to involve negligible energy consumption (manual procedures), the bushes are subjected to a preliminary cleaning, by means of compressed air, milling and wet washing in an agitated (gurgling) water tank. The energy consumption associated with the milling process was estimated using models available in the literature [42].

The material is subsequently sieved and dried at 60 °C for 12 h. The associated energy consumption was calculated using Equation (2):

(2) $$E(\mathrm{kWh})=\frac{m\Delta H_{Vap}}{3.60\eta }$$

where η represents the used equipment energy efficiency (assumed equal to 0.75), ΔH_vap_ is the water vaporization enthalpy (2.26 MJ/kg), m is the mass of eliminated water and the factor 3.60 is used to convert energy from MJ units to kWh.

The cleaning water is recovered via microfiltration, whose maximum energy demand (worst-case scenario) is 4.00 kWh/m^3^ of filtrate [27].

Regarding the recycled carbon fibers, three successive milling stages were performed in order to homogenize the fiber size and reduce the residual polyester mass fraction. Polyester-rich bundles were collected from the milling machine filter. Landfill has been considered as a disposal methodology.

**Figure 23:**
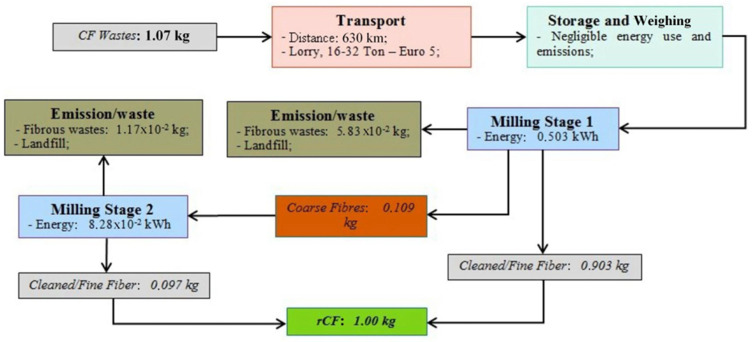
Flow chart regarding the production of 1.00 kg of recycled carbon fibers.

**Figure 24:**
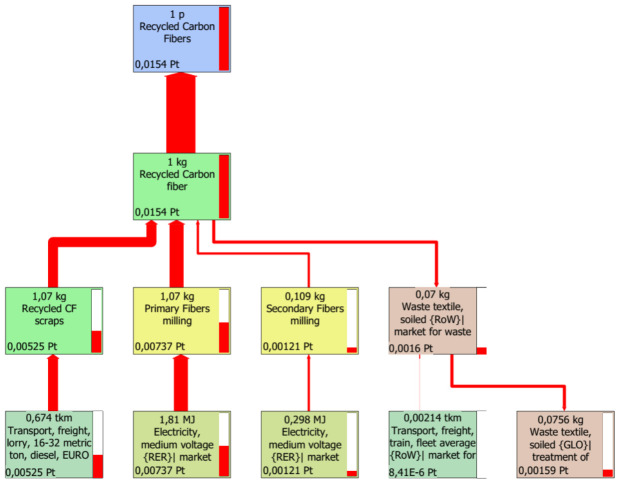
Process tree regarding the production of 1.00 kg of recycled short carbon fibers: 0.50% cut-off.

The flow charts of the recycling processes of high-density polyethylene and the short carbon fibers are schematized in Figure 23 and Figure 24, respectively, while the corresponding inventories are summarized in Tables S2 and S3 in the Supplementary Materials.

The production of the erucamide was also modeled on the basis of literature data [43], as no corresponding dataset is available in the software database. This material is produced through the reaction between fatty acids (specifically erucyl acid) and an excess of ammonia (stirring energy is included [27]), whose unreacted fraction is recovered at the end of the process, involving a limited energy demand, calculated according to Equation (3):

(3) $$Q=\;\frac{m\left(C_{P}\Delta T+\Delta H_{cond}\right)}{\eta }$$

where η represents the process efficiency (assumed equal to 0.65), C_p_ and ΔH_cond_ are respectively the ammonia specific heat and the condensation enthalpy, while ΔT amounts to 150 °C.

Erucyl amide (inventory summarized in Table S3 reported in the Supplementary Materials) and the recycled polyethylene were subsequently compounded via an extrusion process, modeled from the datasets available in the software. Also, in this case, an averaged scenario was built up, whereby 1.00 kg of extruded material was modeled according to the following approach:

- Extrusion, plastic film {RER} Extrusion, plastic film|Cut-off, S:50%;
- Extrusion, plastic pipes {RER} Extrusion, plastic pipes|Cut-off, S:50%.

As introduced, the considered component LCA was carried out by means of the assessment tool ReCiPe 2016(H/A) end-point and ReCiPe 2016(H/A) mid-point. The former allows obtaining a general framework of the environmental impact of the investigated materials, while the second one allows estimating the different impact categories from a numerical point of view.

The recycling route leading to the production of short carbon fibers for use as a reinforcement in thermoplastic polymers is potentially characterized by a very low environmental impact, as shown in Figure 24. In this regard, the calculated LCA score is 1.54 × 10^−2^ Pt, indicating a limited overall potential impact. It should be pointed out that the assessed scenario is based on a hybrid approach, combining both experimental measurements with averaged models. This aims at attempting the upscaling of a laboratory process to industrial-scale operations, typically characterized by a lower energy consumption and scrap rationalization [44,45]. The represented scenario is far from the most favorable one from an environmental point of view, thus relevant improvements can be reasonably expected. Transport is based on primary, measured data, subjected to significant variability depending on the specifically investigated scenario (site-specific parameters).

The process tree represented in Figure 24 shows that the most important phase is the milling process, as it is responsible for about 48% of the total LCA score, followed by transportation, which provides an additional contribution of 34%. The milling process is mainly characterized by energy consumption and represents a potential environmental hotspot, which can be mitigated through the use of industrial-sized equipment and facilities, typically associated with improved energy efficiency.

**Figure 25:**
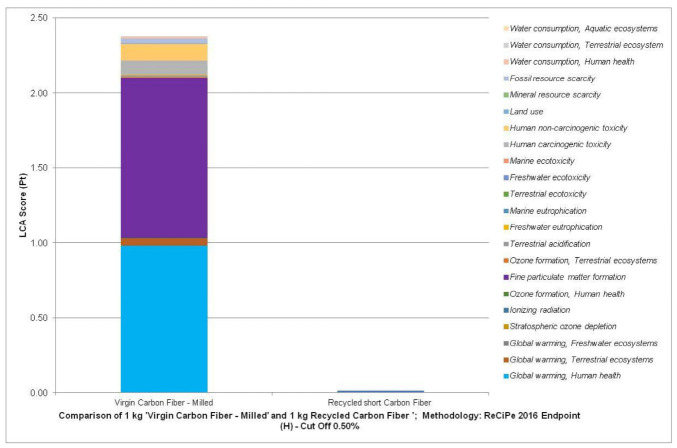
Comparison between the LCA score of virgin and recycled carbon fibers: 0.50% cut-off.

The comparison between the LCA score of virgin short carbon fibers (Figure 25) and those obtained from the recycling process carried out in this study indicates that the recycled fibers exhibit a substantially lower potential environmental impact (about 170 times lower). The described scenario is also supported by the data summarized in Table 15, regarding the values of the impact category indicators for the production of the two competing products.

It should be emphasized that this comparison represents a preliminary assessment of the potential environmental sustainability of the performed recycling chain and does not consider the mechanical performance of the competing products. In this context, virgin (commercial) reinforcements are generally characterized by a narrower fiber length distribution than the recycled fibers. Moreover, the dimensions of the virgin reinforcement prior to the extrusion process probably exceeds the fibers’ critical length, resulting in a more effective strengthening and stiffening performance of the host matrix than the investigated recycled material.

**Figure 26:**
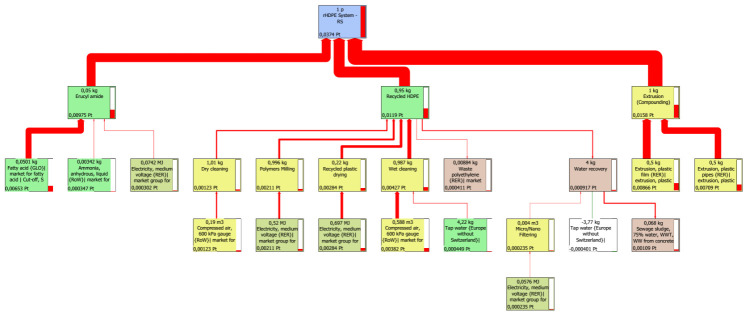
Process tree regarding the production of 1.00 kg of the rHDPE/Erucamide blend: 0.50% cut-off.

The LCA score (Figure 26) of the recycled polyethylene compound is very low (3.74 × 10^−2^ Pt). As in the previous case, lab-scale processes have been modeled and, consequently, further improvements in the environmental performance could be expected at an industrial scale. The most important contribution to the LCA score can be attributed to the extrusion process, which is responsible for 42% of the total impact. This process was modeled through existing averaged datasets and, consequently, no significant reduction in the environmental impact can be expected through further model updating.

Despite being used in a relatively small amount (about 5% in weight), erucamide’s contribution is very important, as it represents 26.10% of the total LCA score. Replacing erucamide with alternative additives characterized by a lower potential environmental impact could provide an additional enhancement in the overall sustainability of the blend, provided that this substitution does not adversely affect the system workability and properties. The core segment of the supply chain regards the recycling phases and, although based on lab-scale processes, contributes only 32% of the total LCA score. In this case as well, minor improvements can be achieved through an industrial upscaling.

**Figure 27:**
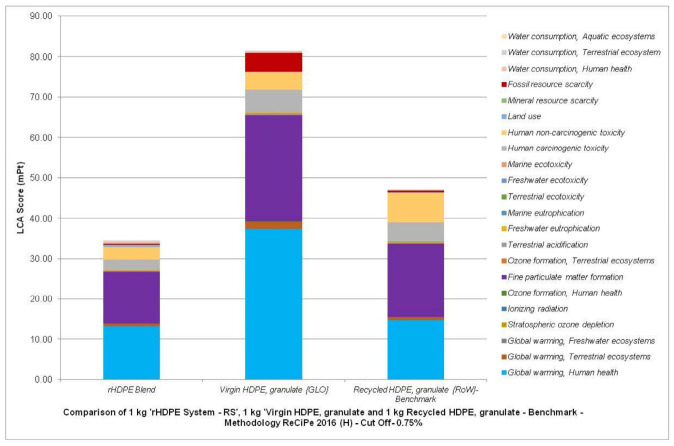
Comparison between the LCA score of virgin, benchmark recycled polyethylene and the developed baseline blend:0.50% cut-off.

The potential environmental impact of the produced blend is in the same order of magnitude as that of the benchmark recycled high-density polyethylene, as shown in Figure 27 and summarized in Table 16, and is considerably lower than that of the benchmark virgin system. These results provide an encouraging preliminary indication of the effectiveness of the recycling route and its environmental sustainability.

For both typologies of materials, a worst-case scenario was considered with respect to waste generation and energy consumption. Consequently, any improvements in material optimization or process scale-up are expected to result in enhanced environmental performance. As introduced, the flows regarding the sleepers’ initial transport are based on site-specific data, which may be subjected to significant variability. A similar consideration applies to the recycling of carbon fibers. Therefore, the sensitivity of the environmental performance of these two key products was analyzed with respect to variations in transport distances.

**Figure 28:**
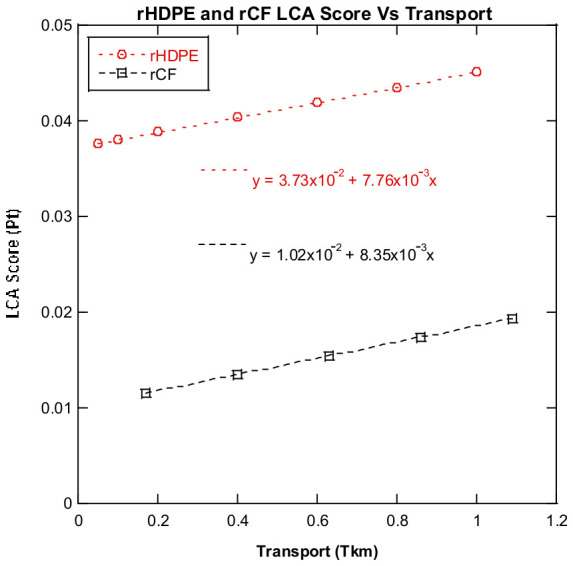
Variation of the recycled HDPE and carbon fiber LCA score with the increase in the amount of transport-based flows.

Figure 28 shows the variation in the LCA score for both typologies of materials as a function of the feedstock initial transport. As expected, the LCA score for recycled polyethylene exhibits minimal variation, consistent with the results previously presented in the process tree shown in Figure 26. Specifically, the variation corresponds to 0.12% of the LCA score per Tkm. In contrast, the LCA score for recycled carbon fiber is substantially influenced by changes in the initial transport, in agreement with the scenario depicted in the process tree of Figure 26. In this case, the variation amounts to 12.12% per Tkm.

In line with the described scenario, for the recycled polyethylene, the impact indicators for recycled polyethylene showed no significant variation with respect to the initial transport, as summarized in Table 17. In contrast, most of the impact indicators for recycled carbon fibers were substantially influenced by variations in transport. The only exception was the indicator related to the ionizing radiation category, which exhibited negligible dependence. Several indicators, including global warming, ozone formation, marine ecotoxicity, land use, and mineral and fossil resource scarcity, displayed variations exceeding 10% per Tkm. Notably, the terrestrial ecotoxicity category showed the most pronounced effect, with a variation exceeding 73% per Tkm.

To conclude the evaluation of the recycling pathway’s sustainability, the costs associated with the production of recycled carbon fibers were estimated. The analysis considered only scrap fibers as input, attributing no economic value to the feedstock.

Cost estimation was based on inventory data from the material’s life cycle assessment (LCA) and included the following categories:

- Transportation: In Italy, full truckload transport costs range from 0.90 to 1.50 €/ton·km, whereas less than full truckload shipments cost between 1.50 and 3.00 €/ton·km. The latter scenario was adopted.
- Energy: Medium-voltage electricity was assumed at an average cost of 0.142 €/kWh.
- Equipment depreciation: Medium to low production rate milling machines were considered, with purchase prices of €50,000–100,000, a service life of 10 years, and annual operation of 5000 h. Carbon fiber milling rates typically range from 40 to 80 kg/h.
- Waste disposal: Landfilling of non-hazardous special waste, such as polyester fiber residues, was included, with costs ranging from 100 to 250 €/t (ISPRA, 2023–2024).

The depicted framework is summarized in Table 18 and indicates a production cost ranging from 1.00€/kg to 1.93 €/kg. Regarding the carbon fiber production cost, literature models [46] report a minimum production cost of 6.89 $/kg (about 6.32 €/kg). The estimated cost is also lower than the cost of recycled carbon fibers sourced from carbon-fiber-reinforced plastic components [47], produced by matrix thermal or chemical degradation.

Furthermore, the estimated cost of recycled fibers is lower than that of recycled carbon fibers recovered from CFRP components [47], which are obtained through thermal or chemical matrix degradation.

These results indicate that the proposed pathway provides a sustainable option in terms of material recovery and potentially offers economic advantages compared to conventional carbon fiber production and alternative recycling routes regardless of the material performance.

## 4. Conclusions

In this study, high-density polyethylene (HDPE) derived from bushes embedded in railway sleepers was successfully recycled through an industrially scalable and environmentally sustainable process, as confirmed by life cycle assessment (LCA). In parallel, a mechanical recycling route was applied to carbon fiber waste, enabling the valorization of both post-consumer and post-industrial streams. The LCA was conducted using a functional unit of 1 kg, focusing on a baseline blend composed of 95 wt% recycled HDPE and 5 wt% erucamide, as well as on recycled carbon fibers. Recycled HDPE was benchmarked against virgin and database recycled materials, while recycled carbon fibers were compared with virgin counterparts. The results showed that recycled carbon fibers exhibited significantly lower environmental impact, mainly due to the avoidance of energy-intensive production. Within their supply chain, milling contributes approximately 50% of the impact, while transportation accounts for 34%. The baseline HDPE blend displayed an environmental impact comparable to, and slightly lower than, benchmark HDPE, confirming the effectiveness of the recycling process. Extrusion represents the main environmental hotspot, while erucamide contributes 26% of the total impact despite being only 5 wt% of the formulation. The recycling phase itself accounts for approximately 32%. Even under worst-case assumptions, transport variations have negligible influence on HDPE and only moderate effects on recycled fibers. From an economic perspective, recycled carbon fibers show a clear advantage, with a maximum cost of 1.93 €/kg compared to approximately 6.32 €/kg for virgin fibers. From a materials viewpoint, the recycled HDPE exhibited high viscosity, heterogeneity, and poor toughness, requiring the use of erucamide and a compatibilizer to ensure processability. Recycled carbon fibers showed high purity (96 wt%), although milling generated a wide length distribution. Weibull analysis identified a most probable fiber length of 2.16 mm for milled fibers and 1.76 mm for fibers from filters. The incorporation of carbon fibers significantly improved stiffness, with Young’s modulus increasing linearly with fiber content and exceeding 4.00 GPa for all systems. Tensile strength remained nearly constant for most composites, except in compatibilized systems, where improvements were observed. The maximum increase in yield strength reached 20% at 30 wt% fiber content. Conversely, ductility decreased substantially, with yield strain reductions up to 65%. Systems without compatibilizer showed the lowest mechanical performance due to poor interfacial adhesion, while compatibilized systems achieved the best balance of properties. Carbon fiber addition also enhanced thermal stability and electrical conductivity. Electrical behavior depended strongly on fiber content and interface quality. Composites with 10 wt% fibers remained insulating, indicating a percolation threshold above this value. Systems containing only erucamide became dissipative at 20 wt% and weakly conductive at 30 wt%, while those with both erucamide and compatibilizer exhibited dissipative behavior even at the highest fiber content. Stronger interfacial adhesion corresponded to lower electrical conductivity, while weaker interfaces favored conductive network formation. Thermally, recycled HDPE from both bushes and closures showed typical melting and crystallization temperatures and high stability. Erucamide exhibited lower thermal stability, potentially limiting processing, but this effect was mitigated by fiber addition and by replacing the compatibilizer with recycled polyethylene from bottle closures. The best thermal performance was achieved with 20 wt% recycled closure-derived polyethylene. This substitution resulted in similar stiffness and strain at break, but lower tensile strength and interfacial adhesion, while improving electrical conductivity and ductility.

The obtained results demonstrate that the proposed recycling routes enable the production of multifunctional HDPE-based composites with a favorable balance between environmental sustainability, mechanical reinforcement, thermal stability, and electrical functionality, highlighting the potential of waste-derived polymers and fillers for high-value applications.

## Figures and Tables

**Table 1:** Fibers’ Weibull parameters and average diameter.

Fiber	Weibull x_0_ (mm)	Weibull α	Diameter (μm)
Milled	2.06	2.17	8.87 ± 0.81
Collected from filters	1.53	1.76	7.99 ± 0.23

**Table 2:** Starting materials’ thermal properties.

Material	T_melt_ (°C)	ΔH_melt_ (J/g)	T_cryst_ (°C)	ΔH_cryst_ (J/g)
rHDPE	133.6 ± 3.7	204.0 ± 7.2	116.6 ± 5.2	204.9 ± 6.7
rHDPE__Cap_	132.3 ± 0.7	208.6 ± 0.8	117.1 ± 2.0	208.1 ± 1.9
MB256D	132.0 ± 2.4	202.0 ± 2.5	78.9 ± 1.8	202.1 ± 2.4
			111.7 ± 3.1	
Erucamide	82.9 ± 1.7	87.6 ± 1.9	21.7 ± 0.1	90.2 ± 0.8
			77.0 ± 0.8	

**Table 3:** Neat matrices’ thermogravimetric properties.

Material	T_onset_ (°C)	T_Max_ (°C)	T_end_ (°C)
rHDPE	387.9 ± 1.2	487.8 ± 3.1	514.1 ± 2.1
rHDPE__Cap_	384.1 ± 1.1	492.2 ± 3.0	518.8 ± 3.0
MB256D	378.2 ± 1.2	478.1 ± 2.3	503.1 ± 2.5
Erucamide	202.2 ± 1.1	331.4 ± 1.2	339.4 ± 2.0

**Table 4:** Melt index values for the produced materials and blends.

Material	MFI (g/10 min)	Δ (%)
rHDPE	0.71 ± 0.04	Baseline
rHDPE/Erc	0.77 ± 0.02	+8.45
rHDPE/MB256D	1.36 ± 0.09	+91.55
rHDPE/MB256D/Erc	1.62 ± 0.10	+128.17

**Table 5:** Thermal properties of the produced materials and blends.

Material	rHDPE/Erc	rHDPE/MB256D	rHDPE/MB256D/Erc
*T_melt_*__1_ (°C)	80.7 ± 1.6	-	78.9 ± 1.7
*ΔH_melt_*__1_ (J/g)	5.00 ± 0.1	-	3.4 ± 0.0
*T_melt_*__2_ (°C)	135.4 ± 4.7	134.7 ± 3.4	134.7 ± 3.1
*ΔH_melt_*__2_ (J/g)	209.7 ± 6.8	208.4 ± 7.1	206.7 ± 4.8
T_cryst_1_ (°C)	65.1 ± 1.0	-	62.3 ± 1.0
ΔH_cryst_1_ (J/g)	6.9 ± 0.1	-	3.6 ± 0.1
T_cryst_2_ (°C)	114.7 ± 4.7	115.7 ± 2.9	114.1 ± 4.0
ΔH_cryst_2_ (J/g)	209.4 ± 6.0	213.3 ± 8.0	213.5 ± 5.2

**Table 6:** Thermogravimetric properties of the produced materials and blends.

Material	rHDPE/Erc	rHDPE/MB256D	rHDPE/MB256D/Erc
*T_on_Erc_* (°C)	210.8 ± 1.0	-	202.5 ± 1.1
*T_on_PE_* (°C)	386.4 ± 2.2	388.7 ± 4.0	385.1 ± 5.0
*T_max_Erc_* (°C)	307.4 ± 4.0	-	298.0 ± 3.2
*T_max_PE_* (°C)	476.2 ± 2.6	480.0 ± 2.6	479.9 ± 3.1
*T_end_Erc_* (°C)	352.5 ± 4.2	-	362.5 ± 3.2
*T_end_PE_* (°C)	509.3 ± 3.8	502.3 ± 3.0	508.2 ± 3.0

*T_on_Erc_* and *T_on_PE_*—Onset degradation temperature of erucamide and polyethylene, respectively; *T_max_Erc_* and *T_max_PE_*—Temperature of maximum degradation rate for erucamide and polyethylene, respectively; *T_end_Erc_* and *T_end_PE_*—End degradation temperature for erucamide and polyethylene, respectively.

**Table 7:** Summary of the mechanical properties regarding the developed systems.

Fibers (%_Wt_)	Matrix	σ_Yeld_ (MPa)	ε_Yeld_ (%)	E (GPa)
**0**	rHDPE	49.0 ± 0.5	10.7 ± 0.5	1.7 ± 0.2
	rHDPE/MB256D	44.1 ± 4.1	11.9 ± 1.2	1.7 ± 0.2
	rHDPE/Erc	48.7 ± 2.1	13.6 ± 0.9	1.4 ± 0.1
	rHDPE/MB256D/Erc	36.7 ± 2.1	15.2 ± 1.1	1.2 ± 0.0
**10**	Neat rHDPE	No processability		
	rHDPE/MB256D	48.5 ± 3.8	10.5 ± 1.4	2.2 ± 0.1
	rHDPE/Erc	40.9 ± 2.6	10.1 ± 1.2	2.2 ± 0.1
	rHDPE/MB256D/Erc	46.2 ± 1.8	9.5 ± 1.3	2.8 ± 0.2
**20**	rHDPE	No processability		
	rHDPE/MB256D	49.8 ± 1.2	7.5 ± 0.7	3.5 ± 0.3
	rHDPE/Erc	43.8 ± 2.5	6.1 ± 0.5	3.2 ± 0.2
	rHDPE/MB256D/Erc	47.9 ± 4.6	7.1 ± 1.7	3.3 ± 0.5
**30**	rHDPE	No processability		
	rHDPE/MB256D	53.1 ± 3.2	6.4 ± 1.1	4.1 ± 0.3
	rHDPE/Erc	47.2 ± 1.1	4.7 ± 0.7	4.5 ± 0.1
	rHDPE/MB256D/Erc	50.2 ± 2.4	6.2 ± 1.2	4.4 ± 0.2

**Table 8:** Electrical properties regarding the developed systems.

Material	Fiber (%_Wt_)	ρ_vol_ (Ω * cm)	ρ_sur_ (Ω/sq)	Behavior
HDPE/Erc	0	(4.20 ± 0.28) × 10^16^	(3.77 ± 0.30) × 10^16^	Insulator
	10	(1.55 ± 0.31) × 10^13^	(1.21 ± 0.16) × 10^14^	Insulator
	20	(7.13 ± 0.64) × 10^8^	(3.95 ± 0.59) × 10^7^	Dissipative
	30	(2.90 ± 0.50) × 10^6^	(1.04 ± 0.18) × 10^5^	Conductive
HDPE/MB256D	0	(3.09 ± 0.17) × 10^16^	(2.26 ± 0.38) × 10^16^	Insulator
	10	(2.24 ± 0.38) × 10^16^	(4.10 ± 0.67) × 10^15^	Insulator
	20	(1.63 ± 0.03) × 10^13^	(1.21 ± 0.02) × 10^14^	Insulator
	30	(5.80 ± 0.70) × 10^9^	(3.73 ± 0.39) × 10^10^	Dissipative
HDPE/MB256D/Erc	0	(9.00 ± 0.45) × 10^16^	(4.00 ± 0.31) × 10^17^	Insulator
	10	(3.54 ± 0.44) × 10^16^	(3.01 ± 0.61) × 10^17^	Insulator
	20	(9.62 ± 0.91) × 10^11^	(8.26 ± 0.98) × 10^15^	Dissipative
	30	(3.90 ± 0.36) × 10^9^	(6.25 ± 0.99) × 10^8^	Dissipative

**Table 9:** Melt Flow Index of the blends containing PE from closures.

PE from Caps (%_wt_)	MFI (g/10 min)
0	0.77 ± 0.02
10	1.68 ± 0.12
20	1.87 ± 0.09
30	2.74 ± 0.09
100	6.53 ± 0.11

**Table 10:** Thermal properties of the produced materials and blends.

Material	rHDPE90/PE__Cap_10	rHDPE80/__Cap_20	rHDPE30/__Cap_30
*T_melt__*_1_ (°C)	79.5 ± 1.0	79.2 ± 1.3	80.1 ± 2.1
*ΔH_melt_*__1_ (J/g)	6.9 ± 0.3	6.4 ± 0.4	8.21 ± 0.4
*T_melt_*__2_ (°C)	132.9 ± 1.9	132.8 ± 2.3	132.7 ± 3.0
*ΔH_melt_*__2_ (J/g)	210.8 ± 4.1	208.9 ± 6.8	207.2 ± 5.1
T_cryst_1_ (°C)	65.2 ± 0.5	65.9 ± 0.2	62.1 ± 0.8
ΔH_cryst_1_ (J/g)	6.5 ± 0.4	1.3 ± 0.1	7.3 ± 0.2
T_cryst_2_ (°C)	116.3 ± 1.8	117.1 ± 3.0	117.0 ± 3.9
ΔH_cryst_2_ (J/g)	211.0 ± 3.2	211.8 ± 6.1	211.8 ± 5.0

**Table 11:** Thermogravimetric properties of the produced HDPE/PE-based blends.

Material	PE__Cap_	rHDPE90/PE__Cap_10	rHDPE80/PE__Cap_20	rHDPE70/PE__Cap_30
*T_on_Erc_* (°C)	210.8 ± 1.0	216.5 ± 0.5	222.2 ± 1.3	239.9 ± 1.1
*T_on_PE_* (°C)	386.4 ± 2.2	399.5 ± 0.7	402.8 ± 0.7	418.0 ± 1.5
*T_max_Erc_* (°C)	307.4 ± 4.0	323.6 ± 3.0	367.1 ± 4.2	367.1 ± 4.2
*T_max_PE_* (°C)	476.2 ± 2.6	494.5 ± 2.6	497.9 ± 3.0	495.8 ± 2.2
*T_end_Erc_* (°C)	352.5 ± 4.2	379.3 ± 6.1	378.0 ± 3.0	378.9 ± 3.0
*T_end_PE_* (°C)	509.3 ± 3.8	513.1 ± 3.0	515.0 ± 2.1	515.2 ± 2.0

**Table 12:** Mechanical properties of the produced HDPE/PE-based blends.

PE__Cap_ (%_wt_)	σ_Yeld_(MPa)	ε_Yeld_ (%)	E (GPa)
0	48.7 ± 2.1	13.6 ± 1.0	1.4 ± 0.1
10	46.7± 5.5	14.0 ± 2.8	1.6 ± 0.1
20	44.5 ± 3.6	15.0 ± 3.7	1.4 ± 0.1
30	36.0 ± 3.1	17.1 ± 3.0	1.2 ± 0.1

**Table 13:** Mechanical properties of the reinforced HDPE/PE-based blends.

Fiber (%_Wt_)	σ_Yeld_ (MPa)	ε_Yeld_ (%)	E (GPa)
0	44.5 ± 3.6	15.0 ± 3.7	1.4 ± 0.1
10	34.6 ± 0.9	13.6 ± 1.3	2.8 ± 0.1
20	39.3 ± 2.5	9.4 ± 1.4	3.6 ± 0.3
30	44.6 ± 1.5	8.6 ± 1.6	4.4 ± 0.4

**Table 14:** Electrical properties regarding the reinforced PE blends.

Fibers (%_wt_)	ρ_volume_ (Ω * cm)	ρ_surface_ (Ω/sq)	Behavior
0	(4.20 ± 0.28) × 10^16^	(3.77 ± 0.30) × 10^16^	Insulator
10	(2.56 ± 0.34) × 10^15^	(6.80 ± 0.16) × 10^14^	Insulator
20	(7.51 ± 0.30) × 10^9^	(7.23 ± 0.38) × 10^8^	Dissipative
30	(2.03 ± 0.11) × 10^7^	(1.01 ± 0.02) × 10^5^	Dissipative/Weakly Conductive

**Table 15:** Impact indicators regarding the production of 1.00 kg of virgin and recycled carbon fibers.

Impact Category	Unit	Recycled Carbon Fiber	Virgin Carbon Fiber
Global warming	kg CO_2_ eq	0.40	63.30
Stratospheric ozone depletion	kg CFC11 eq	1.04 × 10^−6^	4.01 × 10^−5^
Ionizing radiation	kBq Co-60 eq	0.13	5.36
Ozone formation, Human health	kg NO_x_ eq	1.00 × 10^−3^	0.15
Fine particulate matter formation	kg PM2.5 eq	4.31 × 10^−4^	0.10
Ozone formation, Terrestrial ecosys.	kg NO_x_ eq	1.04 × 10^−3^	0.15
Terrestrial acidification	kg SO_2_ eq	1.06 × 10^−3^	0.19
Freshwater eutrophication	kg P eq	2.00 × 10^−4^	1.87 × 10^−2^
Marine eutrophication	kg N eq	3.50 × 10^−5^	2.00 × 10^−3^
Terrestrial ecotoxicity	kg 1,4-DCB	2.36	44.44
Freshwater ecotoxicity	kg 1,4-DCB	1.29 × 10^−2^	0.88
Marine ecotoxicity	kg 1,4-DCB	1.81 × 10^−2^	1.19
Human carcinogenic toxicity	kg 1,4-DCB	2.05 × 10^−2^	1.72
Human non-carcinogenic toxicity	kg 1,4-DCB	0.38	29.07
Land use	m^2^a crop eq	1.15 × 10^−2^	0.66
Mineral resource scarcity	kg Cu eq	7.43 × 10^−4^	3.20 × 10^−2^
Fossil resource scarcity	kg oil eq	0.10	18.15
Water consumption	m_3_	4.03 × 10^−3^	0.43

**Table 16:** Impact indicators regarding the production of 1.00 kg of virgin, recycled benchmark polyethylene and the developed baseline blend.

Impact Category	Unit	rHDPE Blend	rHDPE—Bench.	Virgin HDPE
Global warming	kg CO_2_ eq	0.86	0.95	2.41
Stratospheric ozone depletion	kg CFC11 eq	8.14 × 10^−7^	2.80 × 10^−7^	3.57 × 10^−7^
Ionizing radiation	kBq Co-60 eq	0.22	2.94 × 10^−2^	5.84 × 10^−2^
Ozone formation, Human health	kg NO_x_ eq	1.34 × 10^−3^	2.60 × 10^−3^	5.21 × 10^−3^
Fine particulate matter formation	kg PM2.5 eq	1.23 × 10^−3^	1.73 × 10^−3^	2.50 × 10^−3^
Ozone formation, Terrestrial ecosys.	kg NO_x_ eq	1.40 × 10^−3^	2.69 × 10^−3^	5.61 × 10^−3^
Terrestrial acidification	kg SO_2_ eq	4.01 × 10^−3^	2.66 × 10^−3^	6.38 × 10^−3^
Freshwater eutrophication	kg P eq	4.59 × 10^−4^	9.87 × 10^−4^	4.29 × 10^−4^
Marine eutrophication	kg N eq	2.64 × 10^−4^	2.12 × 10^−4^	3.49 × 10^−5^
Terrestrial ecotoxicity	kg 1,4-DCB	1.75	6.52	5.13
Freshwater ecotoxicity	kg 1,4-DCB	3.92 × 10^−2^	8.92 × 10^−2^	5.40 × 10^−2^
Marine ecotoxicity	kg 1,4-DCB	4.63 × 10^−2^	0.12	7.18 × 10^−2^
Human carcinogenic toxicity	kg 1,4-DCB	4.55 × 10^−2^	8.46 × 10^−2^	0.10
Human non-carcinogenic toxicity	kg 1,4-DCB	0.81	1.97	1.18
Land use	m^2^a crop eq	0.23	2.97 × 10^−2^	2.08 × 10^−2^
Mineral resource scarcity	kg Cu eq	1.86 × 10^−3^	3.56 × 10^−3^	4.10 × 10^−3^
Fossil resource scarcity	kg oil eq	0.16	0.23	1.68
Water consumption	m^3^	2.57 × 10^−2^	3.79 × 10^−3^	7.75 × 10^−3^

**Table 17:** Impact indicators’ variation with transport in the life cycle of 1.00 kg of recycled high-density polyethylene and 1.00 kg of recycled carbon fibers.

Impact Indicator	Recycled CF (%/Tkm)	rHDPE (%/Tkm)
Global warming	12.16	0.25
Stratospheric ozone depletion	1.73	0.12
Ionizing radiation	0.43	1.37 × 10^−2^
Ozone formation, Human health	15.57	0.46
Fine particulate matter formation	9.24	0.14
Ozone formation, Terrestrial ecosystems	16.22	0.47
Terrestrial acidification	7.84	9.25 × 10^−2^
Freshwater eutrophication	1.59	3.49 × 10^−2^
Marine eutrophication	1.17	7.18 × 10^−3^
Terrestrial ecotoxicity	73.04	2.58
Freshwater ecotoxicity	8.84	0.18
Marine ecotoxicity	10.67	0.30
Human carcinogenic toxicity	18.93	0.18
Human non-carcinogenic toxicity	9.86	3.82 × 10^−2^
Land use	19.39	0.27
Mineral resource scarcity	18.83	0.40
Fossil resource scarcity	16.56	1.59 × 10^−2^
Water consumption	2.08	0.25

**Table 18:** Inventory of cost regarding the production of 1.00 kg of recycled carbon fibers.

Flow	Amount (Unit)	Cost/Unit	Total (€)
Transportation	0.589 Tkm	1.50 ÷ 3.00	0.89 ÷ 1.77
Energy	0.589 kWh	0.142	0.09
Devices depreciation	1.07 kg/kg	0.012 ÷ 0.05	0.013 ÷ 0.054
Waste disposal	0.07 kg	0.10 ÷ 0.25	0.007 ÷ 0.018
TOTAL			1.00 ÷ 1.93

## Data Availability

The original contributions presented in this study are included in the article/Supplementary Materials. Further inquiries can be directed to the corresponding author.

## Supplementary Materials

The following supporting information can be downloaded at: https://www.mdpi.com/article/10.3390/polym18111309/s1, Figure S1: DSC thermograms for the reinforced blends—II Heating scan. Figure S2: DSC thermograms for the reinforced blends—Cooling scan. Figure S3: Summary of the trend found for the mechanical properties related to the produced materials and composites. Figure S4: Fiber alignment in the flow direction for the reinforced systems, based on HDPE/Erucamide (part a), HDPE/MD256D (part b) and HDPE/Erucamide/MB256D (part c). Figure S5: Surfacial resistivity of the produced composites. Figure S6: Melt flow index trend with the increase in the mass fraction of PE obtained from closures. Figure S7: DSC thermograms for the reinforced rPE/rHDPE blends—II° Heating scan. Figure S8: DSC thermograms for the reinforced rPE/rHDPE blends—Cooling scan. Figure S9: Weight and DTG variation with the temperature for the PEBushes/PE_Cap_-reinforced blends. Figure S10: Volume and surface resistivity of the reinforced PE blends. Table S1: Thermogravimetric properties of the produced HDPE/PE reinforced blends. Table S2: Inventory related to the production of recycled polyethylene and carbon fibers—1.00 kg. Table S3: Inventory related to the production of Erucamide—1.00 kg.

## Abbreviations

The following abbreviations are used in this manuscript:

LCA	Life cycle analysis
HDPE	High-density polyethylene
rHDPE	Recycled high-density polyethylene
CF	Carbon fiber
rCF	Recycled carbon fibers
PET	Polyethyleneterephthalate
GFRP	Glass-fiber-reinforced plastic
TNT	Textile non-textile
TGA	Thermogravimetric analysis
DSC	Differential scanning calorimetry
Erc	Erucamide
MB256	Fusabond MB256D
PE	Polyethylene
SEM	Scanning electron microscopy

## References

[1] Koohmishi M., Kaewunruen S., He X., Guo Y. (2025). Advancing railway sustainability: Strategic integration of circular economy principles in ballasted track systems. J. Clean. Prod..

[2] Sañudo R., Goswami R.R., Ricci S., Miranda M. (2022). Efficient Reuse of Railway Track Waste Materials. Sustainability.

[3] Potapov D., Vitolberg V., Shumyk D., Ovcharenko V., Bulgakov V. (2018). Reused rails for underground systems. MATEC Web Conf..

[4] Dolci G., Rigamonti L., Grosso M. (2020). Potential for improving the environmental performance of railway sleepers with an outer shell made of recycled materials. Transp. Res. Interdiscip. Perspect..

[5] Zhang D., Gao C., Hao X., Jing G., Zhang X., Wu Y., Li X. (2024). Composite materials using recycled high-density polyethylene plastic for railway sleepers. Emerg. Mater. Res..

[6] Contreras I.N., Bader J., DuRant P., Grafman L. (2018). An Analysis on Recycling High Density Polyethylene with Limited Resources. Int. J. Serv. Learn. Eng. Humanit. Eng. Soc. Entrep..

[7] Gandhi N., Farfaras N., Wang N.-H.L., Chen W.-T. (2021). Life Cycle Assessment of Recycling High-Density Polyethylene Plastic Waste. J. Renew. Mater..

[8] Murat B.I.S., Kamalruzaman M.S., Azman M.H.N., Misroh M.F. (2020). Assessment of Mechanical Properties of Recycled HDPE and LDPE Plastic Wastes. International Conference on Sustainable Materials (ICoSM 2020).

[9] Zhang J., Hirschberg V., Rodrigue D. (2022). Blending Recycled High-Density Polyethylene HDPE (rHDPE) with Virgin (vHDPE) as an Effective Approach to Improve the Mechanical Properties. Recycling.

[10] Spychała M.J., Latko-Durałek P., Miedzinska D., Sałasinska K., Cetnar I., Popławski A., Boczkowska A. (2024). Structural and Mechanical Properties of Recycled HDPE with Milled GFRP as a Filler. Materials.

[11] Ghernaout D., Belaadi A., Bumaaza M., Chai B.X., Jawaid M., Abdullah M.M.S., Krishnasamy P., Al-Khawlani A. (2024). Effects of incorporating cellulose fibers from *Yucca treculeana* L. on the thermal characteristics of green composites based on high-density poly-ethylene: An eco-friendly material for cleaner production. J. Mater. Res. Technol..

[12] Ibrahim I., Mubarak S., Ashour A., Zeiada W., Radwan A. (2026). Innovative Sustainable Composites: Enhancing Recycled HDPE Properties with Waste Aluminium and Carbon Fiber Additives. Polym. Compos..

[13] Borkar A., Hendlmeier A., Simon Z., Randall J.D., Stojcevski F., Henderson L.C. (2022). A comparison of mechanical properties of recycled high-density polyethylene/waste carbon fiber via injection moulding and 3D printing. Polym. Compos..

[14] Hsieh C.-T., Pan Y.-J., Lin J.-H. (2017). Polypropylene/High-Density Polyethylene/Carbon Fiber Composites: Manufacturing Techniques, Mechanical Properties, and Electromagnetic Interference Shielding Effectiveness. Fibers Polym..

[15] Isa A., Nosbi N., Ismail M.C., MdAkil H., Ali W.F.F.W., Omar M.F. (2022). A Review on Recycling of Carbon Fibres: Methods to Reinforce and Expected Fibre Composite Degradations. Materials.

[16] Dong C., Li K., Jiang Y., Arola D., Zhang D. (2018). Evaluation of Thermal Expansion Coefficient of Carbon Fiber Reinforced, Composites Using Electronic Speckle Interferometry. Opt. Express.

[17] Kumar N., Gangwar A.K., Devi K.S. (2018). Carbon Fibers in Biomedical Applications. Recent Developments in the Field of Carbon Fibers.

[18] Meng F., Olivetti E.A., Zhao Y., Chang J.C., Pickering S.J., McKechnie J. (2018). Comparing Life Cycle Energy and Global Warming Potential of Carbon Fibre Composite Recycling Technologies and Waste Management Options. ACS Sustain. Chem. Eng..

[19] Bledzki A.K., Seidlitz H., Goracy K., Urbaniak M., Rösch J.J. (2021). Recycling of Carbon Fiber Reinforced Composite Polymers—Review—Part 1: Volume of Production, Recycling Technologies, Legislative Aspects. Polymers.

[20] Bhandari M., Nam I.W. (2024). A Critical Review on the Application of Recycled Carbon Fiber to Concrete and Cement Composites. Recycling.

[21] Meng F., Mckechnie J., Pickering S.J. (2018). An Assessment of Financial Viability of Recycled Carbon Fiber in Automotive Applications. Compos. Part A Appl. Sci. Manuf..

[22] Heidarian P., Mokhtari F., Naebe M., Henderson L.C., Varley R.J. (2024). Reclamation and reformatting of waste carbon fibers: A paradigm shift towards sustainable waste management. Resour. Conserv. Recycl..

[23] Pakdel E., Kashi S., Varley R., Wang X. (2021). Recent progress in recycling carbon fiber reinforced composites and dry carbon fiber wastes. Resour. Conserv. Recycl..

[24] Al Zahmi S., Alhammadi S., El Hassan A., Ahmed W. (2022). Carbon Fiber/PLA Recycled Composite. Polymers.

[25] Ateeq M. (2023). A state of art review on recycling and remanufacturing of the carbon fiber from carbon fiber polymer composite. Compos. Part C Open Access.

[26] Pearson A., Liao W., Kazemi Y., Duncan M., Kakroodi A., Heydrich M., Hammami A., Slingerland E., Naguib H.E. (2022). Fiber-matrix adhesion between high-density polyethylene and carbon fiber. Polym. Test..

[27] Carpenter K., Getlein J. An Innovative Approach to Controlling Plastic Grinders in Injection-Molding Plants. Proceedings of the 2013 ACEEE Summer Study on Energy Efficiency in Industry.

[28] (2019). Plastics—Determination of Tensile Properties. Part 1: General Principles.

[29] (2011). Plastics—Determination of the Melt Mass-Flow Rate (MFR) and Melt Volume-Flow Rate (MVR) of Thermoplastics.

[30] Kroisová D., Dvorackova S., Bilek M., Skrivanek J., Białkowska A., Bakar M. (2025). Oxidation Process and Morphological Degradation of Drilling Chips from Carbon Fiber-Reinforced Polymers. J. Compos. Sci..

[31] Nazarenko O.B., Melnikova T.V., Visakh P.M. (2016). Thermal and Mechanical Characteristics of Polymer Composites Based on Epoxy Resin, Aluminium Nanopowders and Boric Acid. J. Phys. Conf. Ser..

[32] Guimarães Silva C.V., da Silva Filho E.A., Uliana F., Rangel de Jesus L.F., de Melo C.V.P., Barthus R.C., Rodrigues J.G.A., Vanini G. (2018). PET glycolysis optimization using ionic liquid [Bmin]ZnCl_3_ as catalyst and kinetic evaluation. Polímeros.

[33] Alhulaybi Z., Dubdub I. (2023). Comprehensive Kinetic Study of PET Pyrolysis Using TGA. Polymers.

[34] Özbek Y., Al-Nadhari A., Eskizeybek V., Yıldız M., Şaş H.S. (2025). Influence of Strand Size and Morphology on The Mechanical Performance of Recycled Cf/PEKK Composites: Harnessing Waste for Aerospace Secondary Load-Bearing Applications. Compos. Part B Eng..

[35] Sacchet S., Valentini F., Rizzo C., Po R., Fambri L. (2025). High density polyethylene with phase change materials for thermal energy management. Energy Mater..

[36] Lima P.S., Trocolli R., Wellen R.M.R., Rojo L., Lopez-Manchado M.A., Fook M.V.L., Silva S.M.L. (2019). HDPE/Chitosan Composites Modified with PE-g-MA. Thermal, Morphological and Antibacterial Analysis. Polymers.

[37] Khan S.M., Gull N., Munawar M.A., Islam A., Zia S., Shafiq M., Sabir A., Awais S.M., Butt M.A., Butt M.T.Z. (2016). 2D Carbon Fiber Reinforced High Density Polyethylene Multi-Layered Laminated Composite Panels: Structural, Mechanical, Thermal, and Morphological Profile. J. Mater. Sci. Technol..

[38] Mallick P.K. (2007). Fiber-Reinforced Composites—Materials, Manufacturing and Design.

[39] Thongruang W., Spontak R.J., Balik C.M. (2002). Correlated Electrical Conductivity and Mechanical Propertiey Analysis of High-Density Polyethylene Filled with Graphite and Carbon Fibers. Polymer.

[40] (2006). Environmental Management—Life Cycle Assessment—Principles and Framework.

[41] (2006). Environmental Management—Life Cycle Assessment—Requirements and Guidelines.

[42] Liang J.-Z., Yang Q.-Q. (2017). Effects of carbon fiber content and size on electric conductive properties of reinforced high density polyethylene composites. Compos. Part B.

[43] Weyand S., Kawajiri K., Mortan C., Schebek L. (2023). Scheme for Generating Upscaling Scenarios of Emerging Functional Materials Based Energy Technologies in Prospective LCA (UpFunMatLCA). J. Ind. Ecol..

[44] Hoong S.S., Ahmad S., Hassan H.A. (2006). Process for the Production of Fatty Acidamides.

[45] Ansar M.A., van Zelm R., Ragas A.M.J. (2025). Closing Data Gaps for LCA of Pharmaceutical Production: Estimating Energy Usage by Upscaling Laboratory Data. ACS Sustain. Chem. Eng..

[46] Amaninder S.G., Visotsky D., Mears L., Summers J.D. (2017). Cost Estimation Model for Polyacrylonitrile-Based Carbon Fiber Manufacturing Process. J. Manuf. Sci. Eng..

[47] Shehab E., Meiirbekov A., Amantayeva A., Suleimen A., Tokbolat S., Sarfraz S. (2021). A Cost Modelling System for Recycling Carbon Fiber-Reinforced Composites. Polymers.

