# Supplementary material for: Development and Characterization of Thermoplastic Composites Based on Recycled HDPE from Railway Sleepers’ Fastening Bushes and Scraped Fractions from Carbon Fiber Waste Upcycling
Source: Polymers (Basel). 2026 May 26;18(11):1309. doi: 10.3390/polym18111309 (PMC13259431; doi:10.3390/polym18111309)
Supplement: Supplementary file 1 [file polymers-18-01309-s001.zip › polymers-4249849-supplementary.pdf]

SUPPLEMENTARY MATERIAS

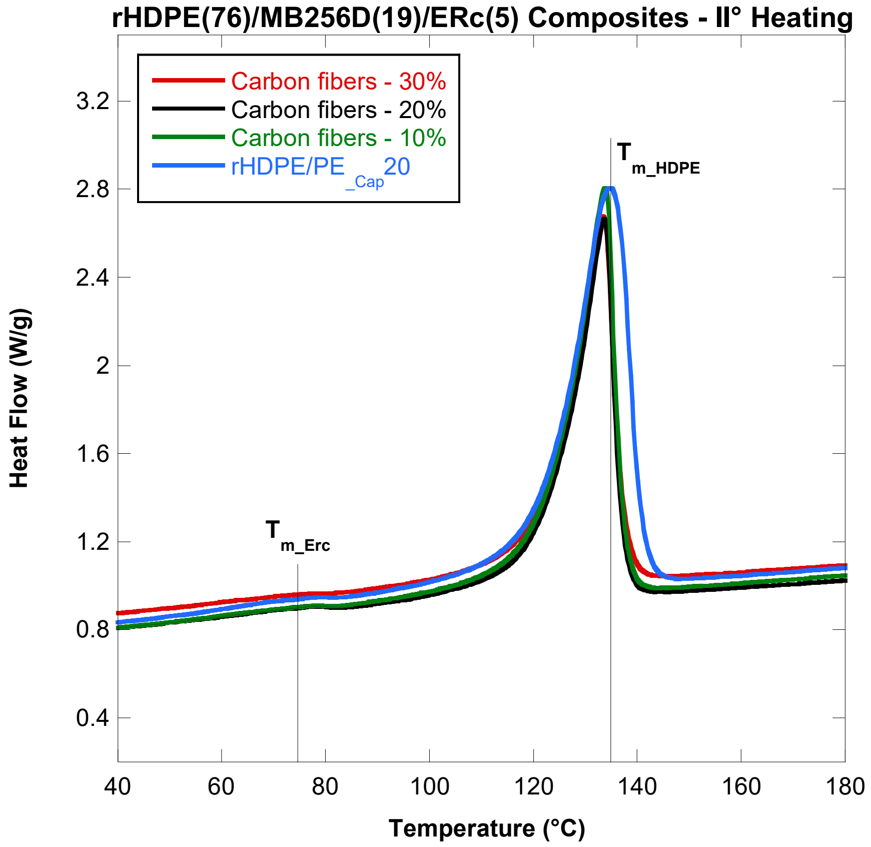

Figure S1: *DSC thermograms for the reinforced blends – II° Heating scan.*

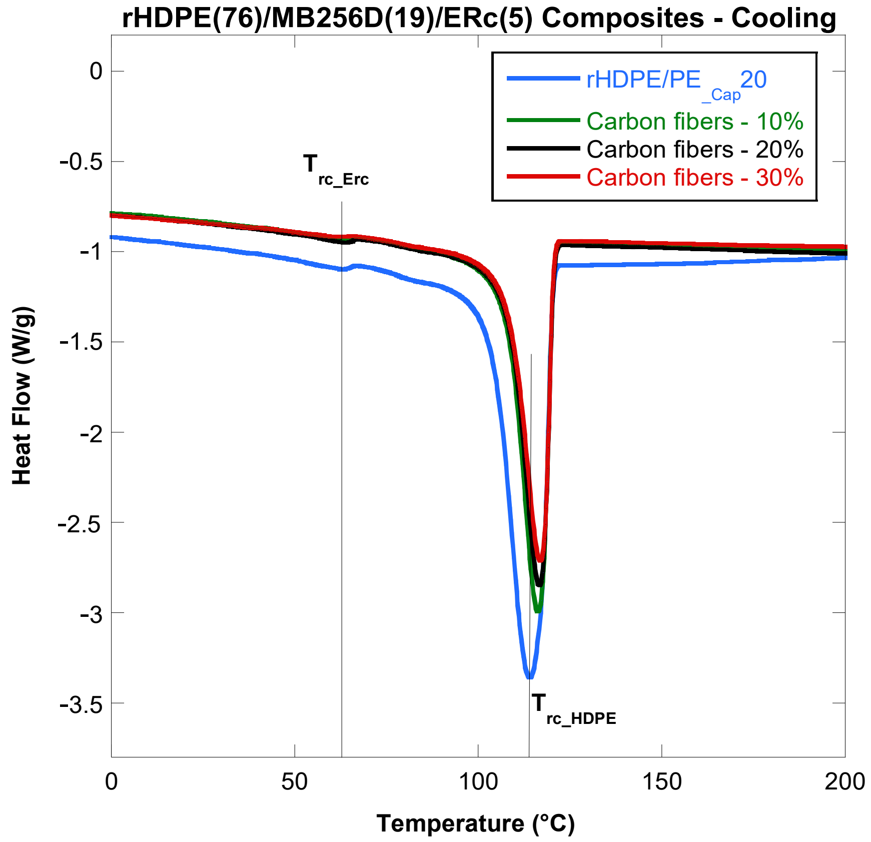

Figure S2: *DSC thermograms for the reinforced blends – Cooling scan.*

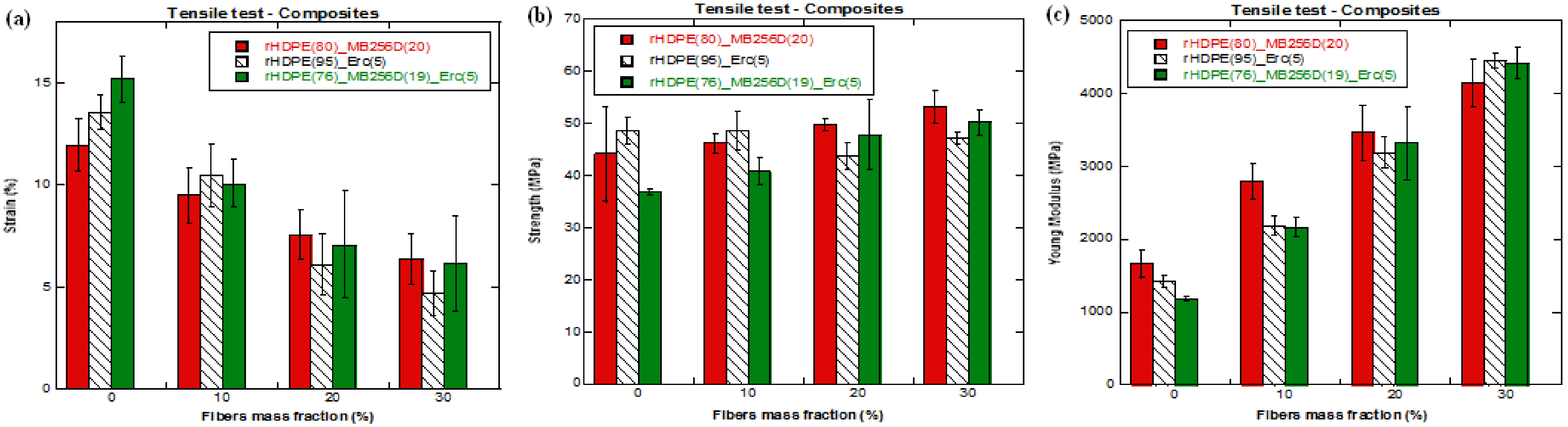

Figure S3: *Summary of the trend found for the mechanical properties related to the produced materials and composites.*

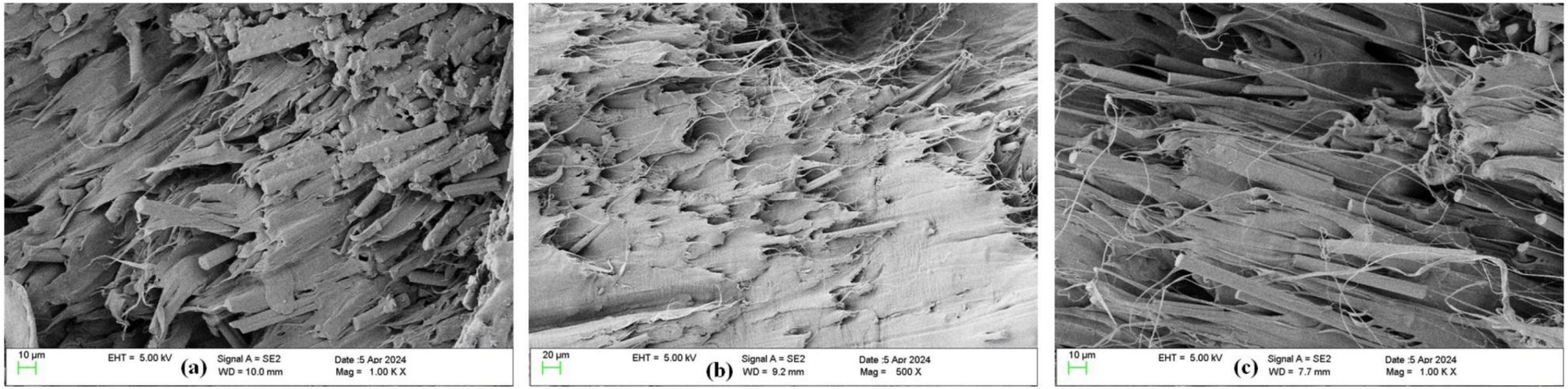

Figure S4: *Fibers allignement in the flow direction for the reinforced systems, based on HDPE/Erucamide (part a), HDPE/MD256D (part b) and HDPE/Erucamide/MB256D (part c).*

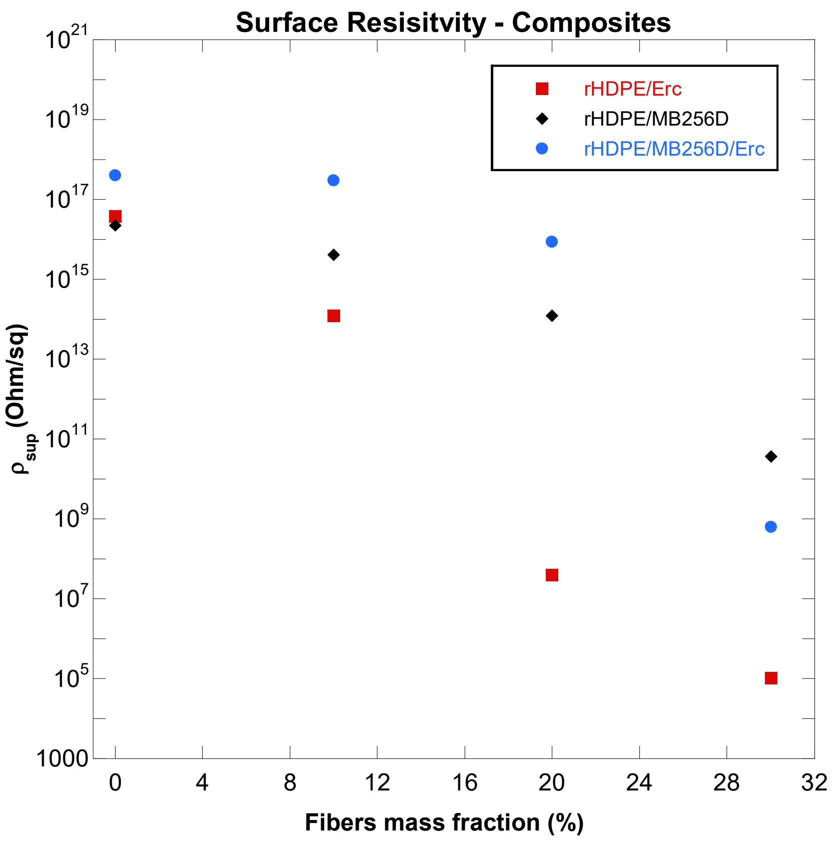

Figure S5: *Surfacial resistivity of the produced composites.*

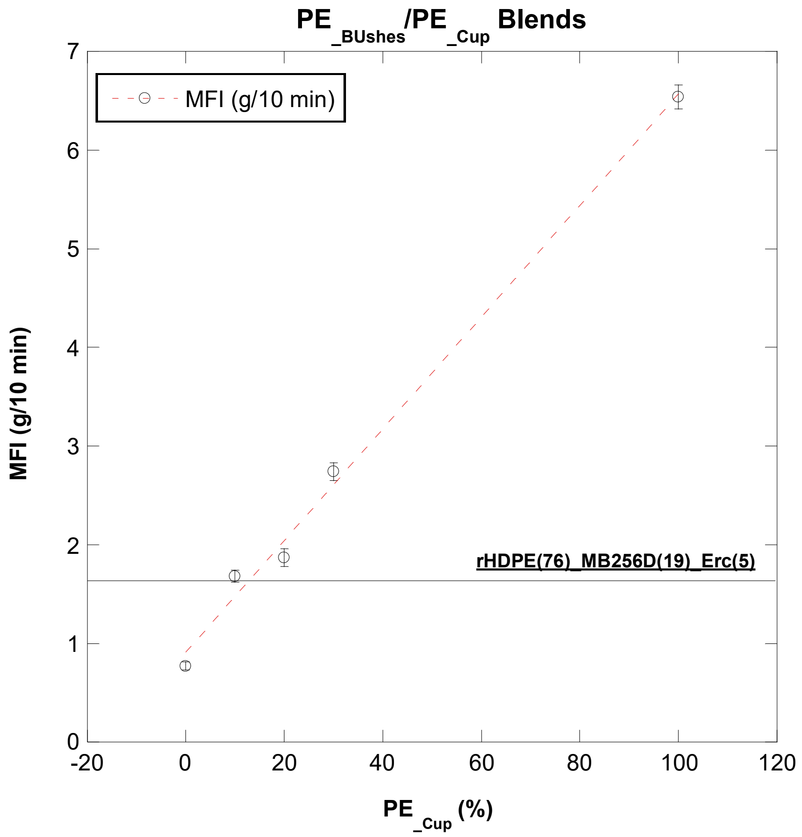

Figure S6: *Melt flow index trend with the increase in the mass fraction of PE obtained from clsosures.*

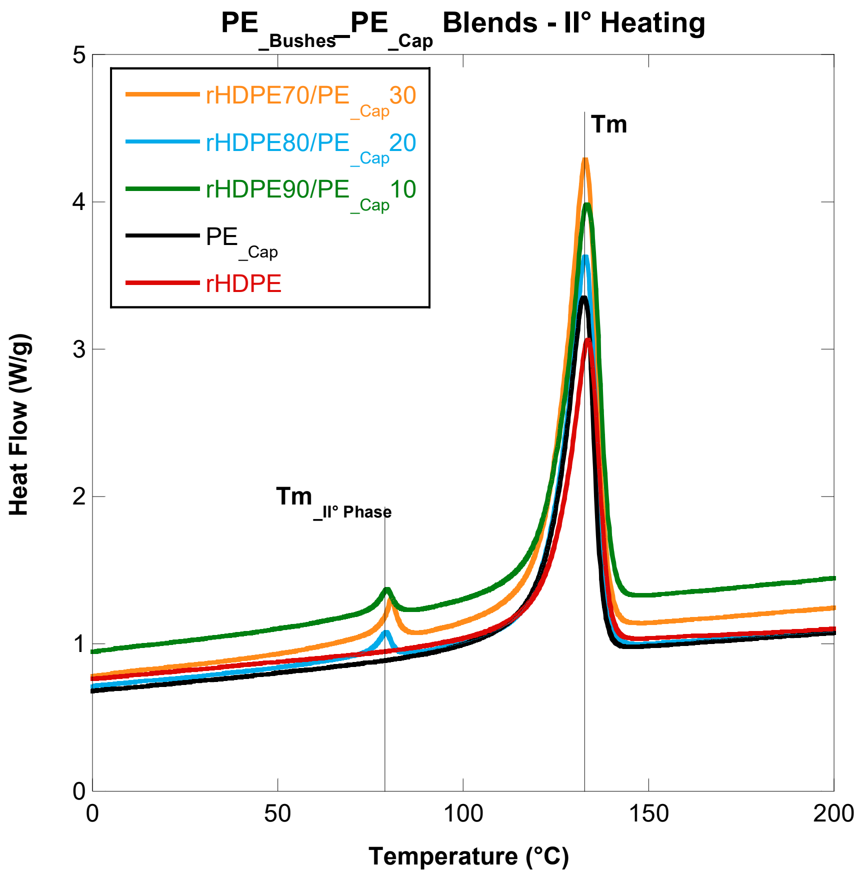

Figure S7: *DSC thermograms for the reinforced rPE/rHDPE blends – II° Heating scan.*

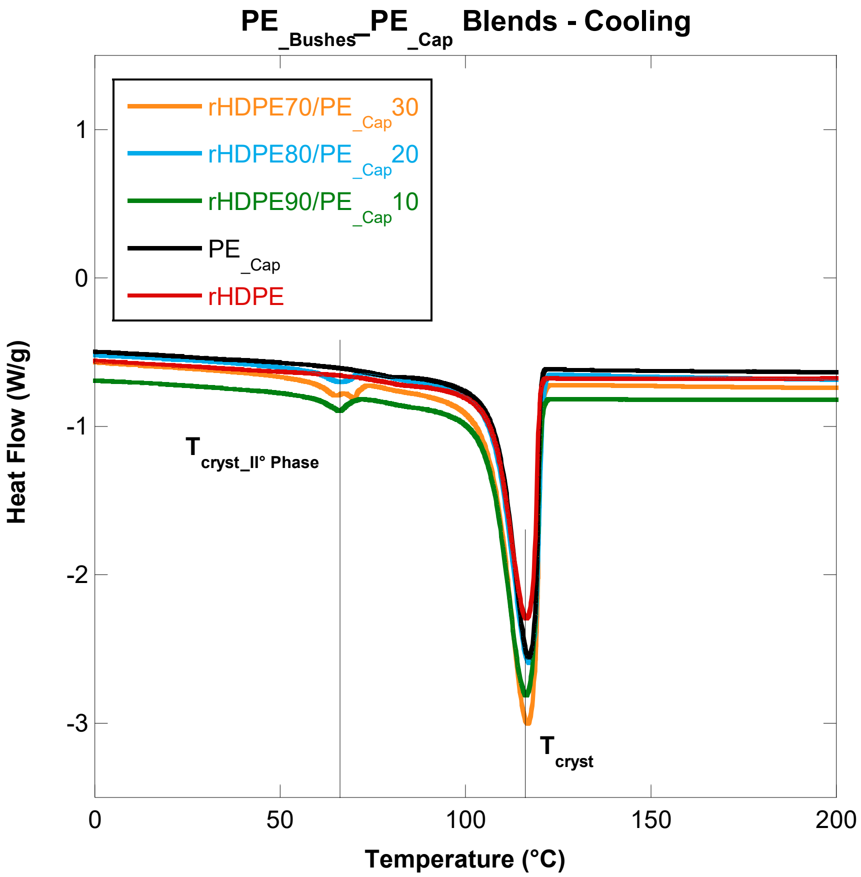

Figure S8: *DSC thermograms for the reinforced rPE/rHDPE blends – Cooling scan.*

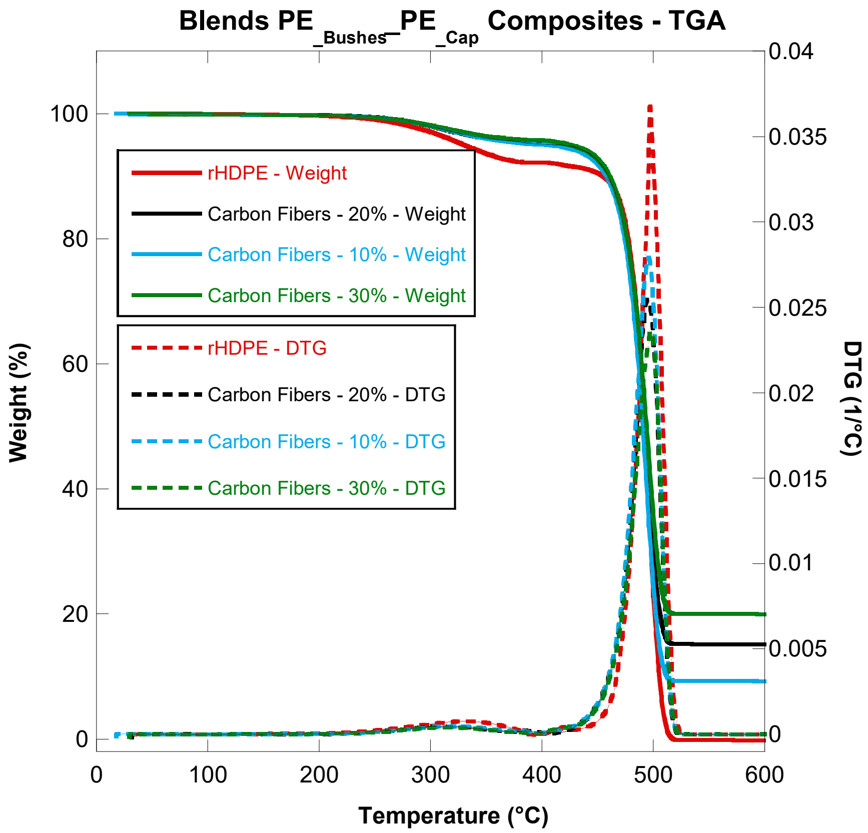

Figure S9: *Weight and DTG variation with the temperature for the PEBushes/PE$_{Cup}$ reinforced blends.*

Table S1: *Thermogravimetric properties of the produced HDPE/PE reinforced blends.*

Fibre (%$_{wt}$)	T_{on_Erc} (°C)	T_{on_PE} (°C)	T_{max_Erc} (°C)	T_{max_PE} (°C)	T_{end_Erc} (°C)	T_{end_PE} (°C)
0	222.2 ± 1.3	402.8 ± 0.7	367.1 ± 4.2	497.9 ± 3.0	37800 ± 3.0	515.0 ± 2.1
10	234.9 ± 1.1	425.0 ± 4.3	324.2 ± 2.4	495.6 ± 3.2	385.8 ± 6.0	514.8 ± 5.0
20	239.0 ± 1.4	428.1 ± 3.0	315.1 ± 1.8	494.9 ± 4.2	392.5 ± 4.2	511.8 ± 3.7
30	233.2 ± 1.1	423.1 ± 1.5	332.2 ± 3.1	497.5 ± 3.3	386.2 ± 3.6	514.1 ± 3.2

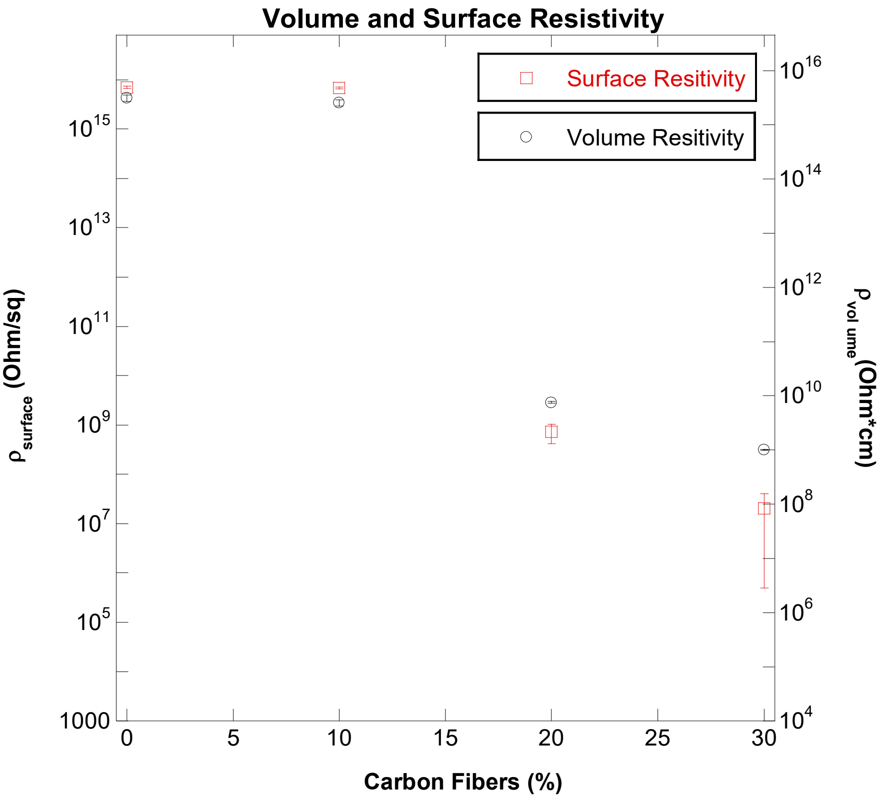

Figure S10: *Volume and surface resistivity of the reinforced PE blends.*

Table S2 Inventory related to the production of recycled polyethylene and carbon fibers – 1.00 kg.

Flow	Unit	rHDPE (1.00 kg)	rCF (1.00 kg)	Dataset/Source
**INPUT**				
Input Plastic bushes	kg	1.052	-	Measuement
Input strips and budles	kg	-	1.07	Measuement
Compressed air	m^3	0.818	-	Compressed air, 600 kPa gauge {RoW}| market for compressed air 600 kPa gauge | Cut-off, S
Tap water (worst case)	m^3	$4.76*10^{-4}$	-	Tap water {Europe without switzerland}| market for tap water | Cut-off, S
Electric energy	kWh	0.348	0.589	Electricity, medium voltage {RER}| market group for electricity, medium voltage | Cut-off, S
Transport	Tkm	$1.05*10^{-2}$	0.59	Transport, freight, lorry,16-32 metric ton, EURO 5 {RER}| market for transport, freight, lorry,16-32 metric ton, EURO 5 | Cut-off, S
**EMISSIONS**				
**In Atmosphere**				
Water vapor	kg	0.232	-	-
**At treatment**				
Landfill	kg	$9.33*10^{-3}$	-	Waste polyethylene {RER}| market group for waste polyethylene | Cut-off, S
	kg	$4.31*10^{-3}$	-	Inert waste, for final disposal{RoW}| treatment of inert waste, inert material landfill | Cut-off, S
	kg	$6.10*10^{-2}$	-	Sewage sludge, 75% water, WWT, WW from concrete production{GLO}| treatment of sewage sludge, 75% water, WWT, WW from concrete production | Cut-off, S
	kg	-	0.07	Waste textile, soiled {RoW}| market for waste textile, soiled | Cut-off, S
Incineration with no energy recovery	kg	$3.70*10^{-2}$	-	Waste polyethylene {RER}| market group for waste polyethylene | Cut-off, S

Wastewater	kg	0.05	-	Wastewater from concrete production{RoW}∣ market for wastewater from concrete production ∣ Cut-off, S

Table S3: Inventory related to the production of Erucamide – 1.00 kg.

**Flow**	**Unit**	**Erucamide**	**Dataset/Source**
**INPUT**			
Erucyl acid	kg	1.002	Measurements
Ammonia	kg	$6.83*10^{-2}$	Ammonia, anhydrous, liquid {RoW}∣ market for ammonia, anhydrous, liquid ∣ Cut-off, S
Butyl chloride hydroxide	kg	$5.01*10^{-3}$	Pesticide, unspecified {GLO}∣ market for Pesticide, unspecified ∣ Cut-off, S
Electric energy	kWh	0.412	Electricity, medium voltage {RER}∣ market group for electricity, medium voltage ∣ Cut-off, S
**EMISSIONS**			
**In Atmosphere**			
Water vapour	kg	$5.03*10^{-2}$	-
Ammonia	kg	$1.78*10^{-2}$	
